# Oral administration microrobots for drug delivery

**DOI:** 10.1016/j.bioactmat.2024.05.005

**Published:** 2024-05-21

**Authors:** An Ren, Jiarui Hu, Changwei Qin, Neng Xia, Mengfei Yu, Xiaobin Xu, Huayong Yang, Min Han, Li Zhang, Liang Ma

**Affiliations:** a State Key Laboratory of Fluid Power and Mechatronic Systems, Zhejiang University, Hangzhou 310058, China; b School of Mechanical Engineering, Zhejiang University, Hangzhou 310058, China; c Department of Mechanical and Automation Engineering, The Chinese University of Hong Kong, Shatin NT, Hong Kong SAR, China; d The Affiliated Stomatologic Hospital, School of Medicine, Zhejiang University, Hangzhou 310003, China; e Key Laboratory of Advanced Civil Engineering Materials of Ministry of Education, Key Laboratory of D&A for Metal-Functional Materials, School of Materials Science & Engineering, Tongji University, Shanghai, 201804 China; f Institute of Pharmaceutics, Zhejiang Province Key Laboratory of Anti-Cancer Drug Research, College of Pharmaceutical Sciences, Zhejiang University, Hangzhou, 310058, China

**Keywords:** Microrobots, Gastrointestinal tract, Oral administration, Drug delivery, Biologics

## Abstract

Oral administration is the most simple, noninvasive, convenient treatment. With the increasing demands on the targeted drug delivery, the traditional oral treatment now is facing some challenges: 1) biologics how to implement the oral treatment and ensure the bioavailability is not lower than the subcutaneous injections; 2) How to achieve targeted therapy of some drugs in the gastrointestinal tract? Based on these two issues, drug delivery microrobots have shown great application prospect in oral drug delivery due to their characteristics of flexible locomotion or driven ability. Therefore, this paper summarizes various drug delivery microrobots developed in recent years and divides them into four categories according to different driving modes: magnetic-controlled drug delivery microrobots, anchored drug delivery microrobots, self-propelled drug delivery microrobots and biohybrid drug delivery microrobots. As oral drug delivery microrobots involve disciplines such as materials science, mechanical engineering, medicine, and control systems, this paper begins by introducing the gastrointestinal barriers that oral drug delivery must overcome. Subsequently, it provides an overview of typical materials involved in the design process of oral drug delivery microrobots. To enhance readers' understanding of the working principles and design process of oral drug delivery microrobots, we present a guideline for designing such microrobots. Furthermore, the current development status of various types of oral drug delivery microrobots is reviewed, summarizing their respective advantages and limitations. Finally, considering the significant concerns regarding safety and clinical translation, we discuss the challenges and prospections of clinical translation for various oral drug delivery microrobots presented in this paper, providing corresponding suggestions for addressing some existing challenges.

## Highlights

• Systematically reviews the advancements of microrobots in oral drug delivery microrobots.
• Provides comprehensive guidelines for designing oral drug delivery microrobots.
• Systematically compares the advantages and limitations of various oral drug delivery microrobots currently available.
• Analyzes the challenges associated with the clinical translation of oral drug delivery microrobots.
• Discusses the prospects of clinical translation for oral drug delivery microrobots.

## Graphical abstract

**Figure:**
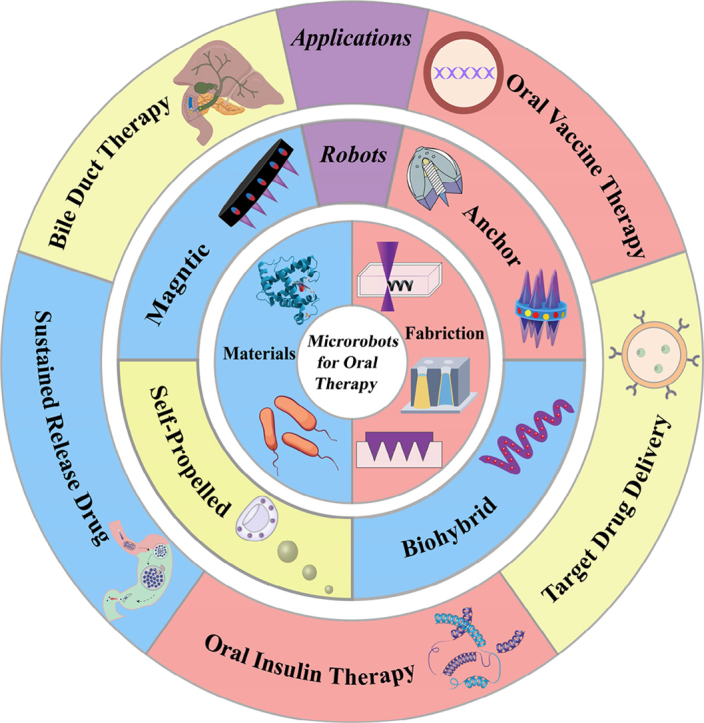

## 1 Introduction

Oral administration refers to the way that the drug formulations are orally delivered to enter the gastrointestinal tract for local or systemic therapy. The oral route is the most common and preferred route for drug administration in recent decades, due to various advantages, such as non-invasiveness, patient compliance and convenience of drug administration [1]. With the continuous development of pharmaceutical technology, biological agents such as proteins and peptides are becoming important components for modern medical therapy. In comparison with the traditional small molecule drugs, biological agents have many advantages, including high activity, high specificity, low toxicity and the minimal nonspecific and drug-drug interactions [2]. These biological agents have become the selection of drugs in certain disease states such as enzyme deficiency, genetic and degenerative disease, as well as protein-dysfunction. However, protein and peptide therapies require parenteral administration. These types of drugs are easily degraded in the gastrointestinal tract, and their relatively large size also limits their transportation through epithelial cells [3]. Moreover, their half-life in serum are usually very short, ranging from a few minutes to a few hours [4]. All these factors lead to ultralow bioavailability of the oral treatment of protein and polypeptide drugs. At present, the most common treatments for these drugs are subcutaneous and intravenous injection, short-term injection may not be an obstacle, but years of daily injections of therapeutic drugs such as insulin may pose challenges to adherence, such as local tissue infections, injection aversion and enetophobia [5]. In addition, a study has shown that needle-phobia, suffered by 10 % of all individuals, may cause certain patients to give up treatment or skip doses [6]. Therefore, the oral route remains the preferred way for drug administration.

**Fig. 1:**
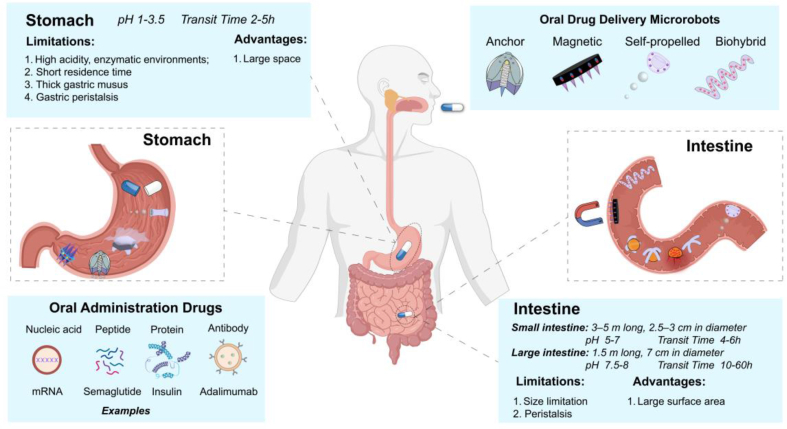
Schematic of the characteristics of the gastrointestinal environment [13,14], and different drugs for oral administration and oral drug delivery microrobots classified by drive mode.

Although the oral administration has clear advantages, drug delivery after administration can be challenging as the complexity and the specific physicochemical features of the human gastrointestinal tract that affect the absorption of the drug molecules especially for biological agents, including varying pH value, cellular and mucus barriers, efflux transporter and metabolic enzymes [1]. Fig. 1 summarizes some characters of the gastrointestinal (GI) environment. So typical problems that always exist in oral administration are poor drug stability, low bioavailability, and low drug permeability of the membrane barriers [7]. As targeted therapy has been more and more important in the modern medical treatment, while routine oral administration is often limited by poor targeting and short cycle times (less than 12 h) [8]. Therefore, how to improve the targeted precision of oral administration is also a problem that needs to be addressed. In recent years, a series of methods have been proposed to solve the existing problems of oral drug therapy [9,10]. To improve the bioavailability of oral delivery drugs, some common methods are applying chemical modifications, absorption enhancers, enzyme inhibitors or bio-adhesive polymers [9,11]. There are some peptides and protein oral formulation that have been approved by FDA, the most famous is the co-formulation of Semaglutide [12], and also this type of glucagon-like peptide-1 (GLP-1) have shown great commercial value, have attracted the attention of many scientific research institutions and enterprises. Though applying this kind of relative traditional pharmaceutical method can realize the oral administration of peptides and protein drugs, the improvement of the bioavailability and the dose is limited. In addition, this type of formulation is fundamentally passive transport in gastrointestinal tract, which to some extent limits the functionality of the drug. Hence active drug delivery with the ability to break through the physiological barriers and targeted releasing still remains a big challenge in oral administration and new technologies are urgently needed.

Microrobots, which are easy to break through various barriers in the gastrointestinal tract and have stronger controllable locomotion ability that can realize active transport of drugs, have attracted extensive attentions in adjuvant drug release. The application of drug delivery microrobots with the capabilities of drug delivery, location and release has been considered as a promising method to solve the problem of oral biological agent administration.

In this paper, the recent progress in oral administration microrobots for drug delivery will be summarized. Based on the different driving modes, the oral administration microrobots can be divided into magnetic-controlled drug delivery microrobots, anchored drug delivery microrobots, self-propelled and biohybrid drug delivery microrobots. We first introduced the gastrointestinal barriers that significantly influence the efficacy of oral drug delivery treatments, providing an explanation of the barriers present in the gastrointestinal tract. Subsequently, in the third section, we presented an overview of materials that may be involved in the design process of oral drug delivery microrobots, then we provided guidelines for designing such microrobots in Section 4, offering a detailed description of various stages to be considered during the design process. Additionally, we outlined some existing solutions to potential issues that may arise at each stage. Different kinds of microrobots are applicable to different scenarios for different tasks (as shown in Fig. 1). This part will be elaborated in Section 5. Considering these oral administration microrobots are still in their early research stage, some studies only showed the microrobot conception has obvious potential of oral drug delivery, but no real drug loaded experiments were carried out, we have concluded the advantages and limitations of different microrobots and discuss the challenges and prospections in clinical translation. We believed that these amazing designs of oral administration microrobots will promote the rapid development of oral drug therapy in the future.

## 2 Gastrointestinal barriers

The primary scenario for oral administration occurs within the gastrointestinal tract, where various barriers present in the gastrointestinal tract are the major obstacles affecting the bioavailability of orally administered drugs. Understanding these gastrointestinal barriers serves as the foundation for designing oral drug delivery microrobots with breakthrough capabilities against gastrointestinal barriers and high bioavailability tailored to different diseases and medications.

### 2.1 Stomach

The stomach, an essential component of the human digestive system, serves as an organ for food storage and digestion, connected to the esophagus above and the duodenum below. The intragastric environment of the stomach is a harsh environment, typically maintaining a pH of approximately 2 in the fasted state. Gastric fluid, a primary biochemical obstacle for drug delivery within the stomach, contains various digestive enzymes such as gastric protease and lipase, capable of breaking down large molecules including proteins [11]. The highly acidic nature of gastric fluid (pH 1–3.5) significantly suppresses the bioavailability of drugs. Consequently, conventional macromolecular drugs administered orally may lose efficacy upon entering the stomach due to its acidic environment and numerous digestive enzymes.

Moreover, compared to intestinal tissues, the stomach wall is thicker, and the contents consist of a mixture of viscous chyme and gastric juice in the fed state, affecting drug dissolution and permeation [11]. The continuous secretion of mucus in the stomach, along with the folds and wrinkles on the stomach surface, further complicates drug delivery within the stomach. Additionally, factors such as gastrointestinal motility, osmotic stress, and the sheer stress of gastric fluid flow may influence the pharmacokinetics of drugs by inducing mechanical degradation of drug molecules or reducing their residence time in the stomach [14].

To achieve effective gastric targeted drug delivery, oral drug delivery microrobots often need to overcome these various physiological barriers such as gastric mucus, pH, and peristalsis. Materials used to construct oral drug delivery microrobots should possess the ability to protect drugs from degradation by gastric mucus and withstand peristalsis while enhancing retention time within the stomach [15]. For intestinal targeted drug delivery, it's also necessary to consider how to overcome the barrier of stomach and ensure that the delivery systems can safely and quickly pass through the stomach and enter the intestine.

### 2.2 Intestine

The intestine consists of the small intestine (3–5 m long, with a diameter of 2.5–3 cm) and the colon (1–5 m long, with an inner diameter of 7 cm), whose continuous structure allows for ample interaction with food boluses and unidirectional flow to eliminate waste [14]. However, the relatively small inner diameter of the intestine, especially in the segment of the small intestine, along with its complex and tortuous tubular structure, increases the risk of foreign body obstruction, leading to complications such as diarrhea, infection, and peritonitis [11,14]. Therefore, the Food and Drug Administration (FDA) has established strict guidelines regarding the size and shape of ingestible tablets and capsules (<22 mm). Compared to the stomach, the small intestine has a higher surface area-to-volume ratio and a longer food clearance time (typically 4-6 h), making it the primary site for digestion, absorption, as well as drug delivery [11]. The thinner and less continuous mucus in the small intestine allows for the absorption of a wider range of molecules when designing oral drugs targeting specific cells, the ability to penetrate mucus and reach the cell surface should be a key consideration.

Furthermore, the intestinal peristalsis is more stable (with varying contraction frequencies along the digestive tract; duodenum, 12 times per minute; colon, 2–10 times per hour), making oral drug delivery more predictable and consistent [15]. While the constant force generated by stable intestinal peristalsis poses a long-term residency challenge for drug delivery robots, this controlled and constant force can also provide reliable and sustained propulsion for microrobots, which is a factor that can be utilized in the design of oral intestinal drug delivery microrobots. Additionally, the pH difference between the small intestine and the stomach can serve as a critical triggering factor for drug release.

## 3 Materials

Materials are a crucial component in the design of drug delivery microrobots, with the functionality of these microrobots relying on appropriate material selection. Materials can generally be categorized as natural and synthetic. Natural materials include some organic compounds and certain natural microorganisms, while synthetic materials encompass organic, inorganic, and hybrid organic-inorganic compounds. Compared to synthetic materials, natural materials offer higher biocompatibility and biodegradability, while synthetic materials tend to exhibit better performance in terms of functionality, such as moldability, mechanical strength, and stability. For oral drug delivery robots, it's challenging to determine which type of material is superior; what matters most is understanding the properties of different materials and selecting suitable ones based on the desired functionalities. Therefore, we provide an elucidation of various natural and synthetic materials and summarize their key characteristics in Table 1.

**Table 1:** Materials and their Key Characteristics.

Materials			Key Characters	Typical Materials	Ref
Natural Materials	Microorganism	Microalgae	Fast growth rate, conducive to large-scale use; Unique flagellar structure; Some strains can survive under harsh conditions such as gastric acid.	Spirulina, Chlorella, Diatom	[18,19,78]
		Yeast	Targeting mechanism for pathogen recognition by phagocytic cells; Unclear mechanism of in vivo transportation.	*Saccharomyces cerevisiae*	[21,22,79]
		Bacteria	Ability to target specific tissues and organs; Improvement of intestinal flora; Spores widely used in oral probiotics.	Bacillus	[23,24]
	Organic Materials	Polysaccharide	Modifiable natural structures, and abundant functional groups facilitate material modification according to requirements.	Hyaluronic Acid, Chitosan	[30,35]
		Protein	Low immunogenicity; tunable mechanical properties.	Silk Fibroin	[41]
Synthetic Materials	Organic Materials	Polymer	Excellent processability; Enhanced physicochemical properties; Good mechanical performance.	PEG, PLA, PLGA, PDMS, PVA	[52,67,80,81]
	Inorganic Materials	Porous silicon nanoparticles;	Porous structure; Can degrade through hydration and hydrolysis in water within a few days.	Mesoporous Silica Nanoparticles	[71,72]
		Metal Oxides	High porosity; tunable pore structure; Abundant metal oxide groups.	Iron oxide	[73]
		Quantum Dots (QDs)	QDs-conjugated drugs exhibit higher physiological, metabolic, and cellular efficacy; Can accumulate in the liver or be phagocytized by immune cells.	Ag2S	[74,75,82]
	Organic and Inorganic Materials	MOFs	Having the potential to promote the development of personalized medicine; Stable physicochemical properties; Remarkable ability to protect complex macromolecules.	ZIF	[76]

### 3.1 Natural materials

Natural materials have a wide range of applications of oral administration, and these materials are also easier to get approved by the Food and Drug Administration (FDA). Here, we divide natural materials into two categories including Microorganism and Organic Natural Materials, and we choose some typical natural materials to introduce their properties and applications.

#### 3.1.1 Microorganisms

##### 3.1.1.1 Microalgae

Microalgae are photosynthetic organisms typically defined as a group of single-celled photosynthetic microorganisms living in aquatic (marine and freshwater) and terrestrial environments. They are abundant on Earth and play a crucial role as one of the primary sources of oxygen. Until recent years, their application as biological entities in the field of biomedicine have gained attention. Many types of microalgae are considered non-toxic, biocompatible, and biodegradable natural materials, such as spirulina [16], chlorella [17], diatoms [18], etc.

The unique morphological characteristics and easily functionalizable surfaces of microalgae enable the binding of diagnostic or therapeutic agents to their surfaces [19]. The motility of microalgae has also inspired the development of biohybrid microrobots, where drug molecules can be non-covalently absorbed onto the surface of microalgae and precisely delivered to target areas through controlled movement. Additionally, the spontaneous fluorescence, phototactic behavior, and biomass production of microalgae can be integrated into the design of novel biohybrid materials with various functionalities. Spirulina platensis (S. platensis, SP), a natural microalga with a length of 200–500 μm and three-dimensional (3D) helical shape, is an edible microorganism that has been mass-produced and developed into dietary supplementations due to its richness in multiple nutrients. The oral administration of this digestible microalga has shown antioxidative, anti-inflammatory effects, and regulation of intestinal microbiota, which would be beneficial to the prevention and treatment of many intestinal diseases [20]. Many researchers have reported the use of SP as a microcarrier to construct oral drug delivery microrobots, and has shown effective improvement in intestinal retention, slow release, anti-inflammatory and other effects [16]. Diatoms are single-celled eukaryotic microalgae with numerous metal-binding sites on their surfaces, exhibiting high adsorption capacity for heavy metals [18]. The surface of diatoms has a unique structure, which provides chemical and physical protection to diatoms and enhances their survival capabilities. This unique structure is composed of a silica shell called a frustule [19]. Additionally, diatoms serve as natural antioxidants [17]. Therefore, diatoms show potential in oral treatments for metal poisoning.

##### 3.1.1.2 Yeast

Yeast, as a single-cell microorganism, serves as an effective derivative drug carrier. Its short growth cycle, fast speed, low cost, and physical and chemical properties such as size (2–4 μm), shape, surface charge, and cell wall composition indicate its potential applications in drug delivery microrobots [21]. Yeast particles (YPs) are mainly composed of β-1,3-glucan, which can be recognized by phagocytic cell surface receptors [22]. Due to the lack of yeast β-glucan-degrading enzymes in the stomach and the acid resistance of yeast β-glucan, yeast can avoid digestion in the stomach and efficiently enter the intestine. Once in the intestine, yeast is phagocytized by M cells (Microfold cell) in the Peyer's patches of the small intestine, enters the lymphatic system, and transports the payload to distant lesion sites through macrophage-mediated trafficking [21]. The direct targeting of pathogen recognition mechanisms by yeast to phagocytic cells provides many possibilities for novel drug delivery strategies in the treatment of chronic inflammation, disease inflammation, and cancer immunotherapy. Currently, some yeast strains have been certified by the FDA as Generally Recognized as Safe (GRAS) and have been used for thousands of years as immune adjuvants and food additives [21]. Yeast-based drug delivery strategies show enormous potential in nanoparticle, small molecule, nucleic acid, and vaccine delivery. Their organic integration and complementary advantages with other drug delivery microrobots further expand the feasibility of application implementation and clinical translation.

##### 3.1.1.3 Bacteria

Bacteria are simple single-celled organisms lacking a cell nucleus or membrane-bound organelles. Some bacterial species, especially some facultative anaerobes such as Salmonella spp. And *Escherichia coli*, are known to actively target specific tissues and organs. Genetically engineered anaerobic bacteria expressing particular proteins could exhibit enhanced uptake by mammalian cells and could be utilized for delivering therapeutic cargoes [23] Besides anaerobic bacteria, spores of Bacillus species such as Bacillus subtilis and Bacillus coagulans are widely used in oral probiotics to improve gut microbiota and enhance immunity. Bacillus spores are dormant life forms resistant to harsh environments such as extreme temperatures and pH levels. These spores can survive and reconstitute in the intestinal tract after oral ingestion, where the outer layer of the spore is degraded. Due to these characteristics, probiotic Bacillus spores serve as oral carriers for delivering various beneficial substances to the intestines. Recombinant Bacillus spores have been employed as carriers to deliver foreign antigens to the gastrointestinal tract, eliciting immune responses. Non-recombinant spores, as oral carriers, deliver foreign molecules to the intestine through covalent coupling and adsorption methods [24]. However, living bacteria-based nanocarriers can not only induce infections but also transfer antibiotic resistance genes to other bacteria within the body. Therefore, caution should be exercised to avoid unnecessary side effects when employing bacteria [23].

#### 3.1.2 Organic natural materials

##### 3.1.2.1 Chitosan

Chitosan is a natural polymer with acceptable properties of biocompatibility, biodegradability, nontoxic, and mechanical stability, which is obtained after deacetylation of chitin [25,26]. Except those basic properties, chitosan also performs some vital properties includes hemostatic, bacteriostatic, anticholestermic and anticarcinogenic [26,27]. However, chitosan also exhibits some problems such as poor solubility in neutral and cell culture medium, which inhibit its usage in these cell-related situations. Fortunately, chitosan can be processed into various shapes and sizes owing to its modifiable structure and abundant functional group, making it suitable for various biomedical applications, such as pharmaceutical, drug delivery as well as tissue engineering [28].

Utilize the strong electrostatic interaction between the carboxyl group of alginates and the amino group of chitosan of chitosan/alginate, chitosan shows great potential in colon-targeted oral drug delivery [29]. Furthermore, using suitable crosslinkers such as tripolyphosphate, genipin and multifunctional aldehydes, and carboxylic [30], to form chitosan hydrogels are capable of designing varieties smart and highly applicable drug delivery carriers, which greatly expands the application range of chitosan [31]. Also chitosan hydrogels can be prepared directly from native chitosan or combined with other polymers to obtain the desired properties, and the hydrogel formed by this way can have better biocompatibility due to there is no introduction of organic solvent or toxic crosslinker [32].

##### 3.1.2.2 Hyaluronic acid (HA)

Hyaluronic acid (HA) is a natural, non-sulphated linear glycosaminoglycan that is an important component of extracellular matrix (ECM), which comprises polyanionic disaccharide units of glucuronic acid and *N*-acetylglucosamine alternatively attached by β-1-3 and β-1-4 glycoside bonds [25,33]. These individual disaccharides bind along to form a coiling chain structure of varied length, with relative molecular masses ranging from 10 kDa to 1000 kDa [34]. The biological effect of HA depend heavily on molecular weight, such as HA with low molecular weight (60–200 kDa) possess immunostimulatory, angiogenic, and pathological activities, while HA with high molecular weight (>500 kDa) associated with anti-angiogenic activity [35]. Though HA has lots of excellent properties in biomedical and pharmaceutical field, its poor stability and short biological half-life drastically limit its biomedical applications [36]. Due to the natural of its chemical structure, HA could be easily subjected to chemical modification to HA derivatives and cross-linking to form HA-based hydrogels with superior properties [37,38]. With such a large range of its molecular weight, HA and its derivatives have been an important natural, biodegradable, and non-immunogenicity materials in the drug delivery [36], tissue engineering [37], and biomanufacturing [39].

##### 3.1.2.3 Silk fibroin

Silk which domesticated from the cocoons of *Bombyx mori* silkworm through sericulture and is composed of silk fibroin and sericin, is one of the strongest fibrous proteins in nature with excellent mechanical, biocompatible, adjustable biodegradable properties, as well as low immunogenicity [40,41]. The unusual combination of high strength and extensibility of silk fibroin is a characteristic unavailable in synthetic materials, so this biomaterial suggests new directions to emulate in the pursuit of novel high-performance, multifunctional applications [42]. Silk fibroin has been used in a variety of biomedical applications including tissue engineering [43], drug delivery [44], 3D bioprinting [45,46] and had been approved as a biomaterial by the FDA in 1993 [47].

As a water-soluble protein, to achieve better use of the various excellent properties of silk fibroin, it's important to transform silk fibroin to form silk-based hydrogels. The main crosslinking approach is physical crosslinking which relies on structural transformation of the fibroin protein chain from random coil to β-sheets, while chemical crosslinking is another important crosslinking approach, which is based on the formation of di-tyrosine bonds to crosslink proteins into multiprotein [48]. Incorporating different crosslinking approaches will influence the final hydrogels properties, for physically crosslinked silk fibroin hydrogels always associated with the high stiffness and crystallinity and result in brittle and hard to handle [41,48].

In summary, natural polysaccharide materials such as chitosan and hyaluronic acid, as well as protein materials like silk fibroin, exhibit excellent biocompatibility, biodegradability, and non-toxicity. Chitosan and hyaluronic acid, due to their modifiable natural structures and abundant functional groups, can be conveniently modified to achieve superior performance tailored to specific requirements. But hyaluronic acid suffers from poor stability and a short biological half-life, while chitosan exhibits low solubility in neutral and cell culture media, which hinders the biomedical applications of these polysaccharides materials. Protein materials offer advantages such as tunable mechanical properties and low immunogenicity. In addition, both protein and polysaccharide natural materials face challenges related to poor processability. To improve processability, natural materials often require certain modifications, which may compromise many of their excellent properties. Therefore, enhancing the processability of natural materials is crucial for oral drug delivery microrobots.

### 3.2 Synthetic materials

#### 3.2.1 Organic synthetic materials

Although natural materials have shown many excellent properties, including good biocompatibility, biodegradability, and tunable physical properties, but with the increasing functional demand, it is difficult for a single natural material that can meet all the needs. Therefore, researchers have used chemistry and other means to synthesize various multifunctional materials which do not exist in nature, such as polyethylene glycol (PEG), methacrylate gelatin (GelMA), Polylactic acid (PLA), poly (ε-caprolactone) (PCL) et al. To a certain extent, it has broadened the development of oral administration and other biomedical fields.

##### 3.2.1.1 PEG

PEG is a kind of polymeric material with unique hydrophilicity and electrical neutrality derived from epoxyethane by ring-opening polymerization or derived from anionic polymerization of ethylene oxide and hydroxyl initiators [49,50]. PEG has become the most commonly used polymer in biomedical research because of its high structure flexibility, biocompatibility, amphiphilicity, and high hydration capacity [51]. PEG can be used in biotechnology therapeutics as a tool to slow down drug clearance and shield protein therapeutics from undesirable immunogenicity [51,52]. PEG units used in drug formulations and consumables generally range between molecular weights of 200–60Da [52]. An important strategy to increase the biomedical applications and the chances for PEG and PEG derive to hit the market is PEGylation. PEGylation is a term used to denote modification of therapeutic molecules by conjugation with PEG. Lots of effects have been made to develop novel strategies for conjugation of PEG with these molecules [[51], [52], [53], [54]]. Till now, PEG and PEG-conjugated drugs have been approved by FDA for human [53].

##### 3.2.1.2 GelMA

GelMA is a gelatin derivative containing a majority of meth-acrylamide groups and a minority of methacrylate [55,56]. It possesses the characteristics of both natural and synthetic materials. The lower cytotoxicity of the methacrylate component and its photocurable properties make GelMA a promising soft material in biomedical engineering [57,58]. Most applications of GelMA are GelMA-based hydrogels, which can be crosslinked by photoinitiated radical polymerization (i.e. under UV light exposure with the presence of a photo-initiator) [55,58,59]. The concentration of reactants, light intensity can significantly affect the physical characteristic of the formed hydrogels [60,61]. GelMA hydrogels closely resemble some essential properties of native extracellular matrix (ECM), because of the presence of cell-adhesion and matrix metalloproteinase (MMP) responsive peptide motifs. With the excellent biological properties and tunable physical characteristics, GelMA hydrogels have a wide range of biomedical applications, such as 3D bioprinting [60,62], tissue engineering [61,63], drug delivery [64], and biomedical microrobot [57] et al. Additionally, lots of efforts have been devoted to combining GelMA-based hydrogels and functional nanomaterials, which aims to endow GelMA with enhanced physical-chemical and biological properties [63].

##### 3.2.1.3 PLA

With good biocompatibility, biodegradability, mechanical strength and processing capacity, PLA has become an important polymer material used in the biomedical fields [65]. Moreover, PLA can be degraded by simple lipid bond hydrolysis without the assistance of enzymes which makes it easy to degrade and expel out of human body [66]. Although PLA has a wide range of applications, it has limitations in some specific conditions. For example, in biomedical applications, PLA is often limited by low degradation rate, low hydrophobicity and low toughness [67]. Fortunately, when mixing with other polymers, relevant properties can be significantly improved [68]. Therefore, various PLA blends have been explored for a variety of biomedical applications, such drug delivery microrobots, microcapsule, and tissue engineering [69,70]. In addition, PLA is also widely used as 3D printing material with good printability. In the oral drug delivery microrobot introduced in this review, PLA can be used as a biocompatible material choice for some parts of the traditional rigid robots, such as the rigid shell, to ensure the robot's biocompatibility and biodegradability.

Furthermore, apart from the aforementioned three kinds of materials, there are many other biocompatible and biodegradable materials, such as Poly (ε-caprolactone) (PCL), Poly (lactic-*co*-glycolic acid) (PLGA) etc. These materials also can be used in oral administration and perform enormous potential applications, so when designing oral drug delivery microrobot, suitable biomaterial can be selected according to different requirements based on applications, so as to ensure the biocompatibility of the robot, to reduce the adverse effect to the human body.

#### 3.2.2 Inorganic synthetic materials

Porous silicon nanoparticles (PSNs) represent a class of inorganic nanomaterials characterized by nanostructured porosity, large pore volume, high specific surface area, biodegradability, and modifiable surface chemistry. Utilizing porous silicon nanoparticles for the encapsulation of Glucagon-Like Peptide-1 (GLP-1) demonstrates outstanding binding efficiency and loading capacity. Despite their significant potential for oral drug delivery, PSNs encounter inherent synthetic challenges. While the preparation of particles is relatively straightforward, issues such as poor control over particle size distribution and pore shape and size due to their top-down synthesis approach persist. Moreover, the mechanisms by which they enhance permeation via paracellular, or transcellular pathways remain unclear. In contrast, mesoporous silica nanoparticles (MSNs) offer further advantages in terms of tunable pore volume, pore size, pore morphology, particle size, and morphology. MSNs can be easily synthesized under mild conditions, providing controlled surface area and pore size. Importantly, both porous and non-porous silica nanoparticles degrade via hydration and hydrolysis in aqueous media within a few days, subsequently forming silicic acid, which is cleared from the kidneys via urine. These characteristics position mesoporous silica as a promising candidate for oral drug delivery [71].

In addition to inorganic silica nanoparticles, metal oxides, as common inorganic compounds, play a crucial role in drug release. Various mesoporous metal oxide nanoparticles, including titanium dioxide, alumina, and iron oxide et al., have been successfully synthesized via templating methods. They predominantly exhibit high porosity, tunable pore structure, abundant metal-oxygen groups, and increased surface area, providing sufficient space for drug loading [72]. Notably, iron oxide nanoparticles can be attracted to tumor cells and deliver drugs via an external magnetic field, minimizing systemic side effects by preventing the release of loaded drugs or therapies in healthy tissues. Furthermore, iron oxide nanoparticles are easily biodegradable, and the degraded iron can be absorbed by endogenous hemoglobin, demonstrating potential applications in vivo [73].

Moreover, various oxide hybrid materials or hybrid inorganic materials can harness the excellent properties of different materials to design multifunctional drug delivery microrobots. Furthermore, quantum dot materials have garnered widespread attention in the field of drug delivery in recent years. Studies have demonstrated that silver sulfide quantum dots exhibit oral bioavailability and can enhance the absorption of metformin and nicotinamide mononucleotide [74]. Conjugating insulin with silver sulfide quantum dots encapsulated in chitosan/glucose polymer shells can produce responsive oral insulin formulations for controlling blood glucose levels without inducing hypoglycemia [75].

#### 3.2.3 Organic and inorganic hybrid synthetic materials

In the design of oral drug delivery microrobots, drug delivery microrobots made of pure inorganic materials typically face biocompatibility issues, while organic materials often require cumbersome conjugation procedures or limitations on drug loading and release. Organic-inorganic hybrid materials, as emerging materials, have shown promising potential in the biomedical field due to their unique properties distinct from pure organic or inorganic materials.

Metal-organic frameworks (MOFs), as classical organic-inorganic hybrid materials, consist of ordered porous structures formed by organic ligands and metal ions under specific conditions. These materials exhibit significant performance in drug design and delivery, including large specific surface area, high porosity, tunable internal pore size, and high drug loading capacity [76]. The flexibility of their customized design allows for the incorporation of different drugs, which is difficult to achieve with traditional drug delivery materials. MOFs also possess stable physicochemical properties and structural forms, meeting the requirements for high stability and uniform dispersion in oral administration. Additionally, the open metal or acid-base sites in MOFs can enhance interactions between drug molecules, thereby achieving controlled and sustained drug release. In recent years, MOFs with different functionalities have been designed for oral drug therapy according to various needs.

Despite the potential of MOFs, there are some challenges in designing and synthesizing MOFs for oral administration. These challenges include requirements for biocompatibility, stability, and drug release control. Moreover, MOFs must withstand the harsh conditions of the gastrointestinal tract and possess optimal physicochemical properties to ensure effective drug delivery [76]. In addition to MOFs, various organic-inorganic nanocomposites have also been developed for oral drug therapy [77].

In summary, synthetic materials offer superior versatility compared to natural materials due to their ability to be synthesized and modified according to various requirements, incorporating functional groups or molecules to impart desired properties. Generally, synthetic materials exhibit outstanding processability, mechanical performance, and enhanced intelligence compared to their natural counterparts, presenting significant potential in the field of microrobotics. There is a growing demand for more intelligent and multifunctional microrobots to address increasingly complex and challenging oral drug delivery therapies. However, synthetic materials often entail complex and cumbersome synthesis processes, along with challenges such as synthesis yield and product purity, leading to higher costs for certain materials. Additionally, the introduction of certain functional groups may compromise the biocompatibility of materials, increasing their biotoxicity, which is particularly concerning for applications such as oral drug delivery microrobots that need to enter the body.

Therefore, future advancements in synthetic materials may require improvements in the synthesis processes to enhance production yield and purity, thereby potentially reducing costs. Addressing issues related to the biotoxicity and biocompatibility of synthetic materials is also crucial. Furthermore, long-term toxicity studies are essential to elucidate potential health hazards associated with synthetic materials, providing reliable data to support the clinical translation of microrobots and facilitating broader applications of synthetic materials in the biomedical field.

## 4 Design guideline

When designing oral drug delivery microrobots targeting specific sites and diseases in the gastrointestinal tract, careful consideration should be given to the entire process of the microrobots from oral ingestion to eventual excretion or degradation in the body. Throughout this process, many challenges arise, including: (1) Drug loading and release, (2) Transportation, (3) Overcoming gastrointestinal barriers, and (4) Fabrication. These issues serve as important guiding principles for the design of oral drug delivery microrobots. Below, we address these problems one by one and provide explanations for some commonly used manufacturing methods.

### 4.1 Drug loading and release

In the design of drug delivery microrobots, drug loading and release are crucial for achieving functional objectives. Rational and reliable drug loading and release modules need to be designed based on different oral therapeutic approaches. Drug loading methods mainly include physical and chemical loading. Physical loading involves embedding drug molecules in the pores of materials or loading drugs through the construction of cavities. Common methods include fabricating drug-loaded microneedles using 3D printing or molding [83], producing drug-loaded hydrogels with materials like GelMA or chitosan [84], coating drugs on specific structures of drug delivery microrobots [85], designing a small cavity to load drugs [86], and utilizing porous materials like mesoporous silica to load drugs [71]. Additionally, some porous or nanomaterials have many binding sites on their surfaces, making them suitable for chemical loading by forming chemical bonds with drug molecules.

Chemical loading involves utilizing specific functional groups on drug molecules to form chemical bonds with functional groups on materials in drug delivery microrobots, thereby achieving drug loading [75]. This loading method typically requires special chemical treatment of drug molecules, and the feasibility of bonding between drug molecules and materials in the design of drug delivery microrobots needs to be considered.

In physical loading methods, there is only physical contact between drugs and delivery microrobots, thus no special treatment of drugs is required, and there is less correlation with the types of drugs to be carried. Therefore, physical loading has stronger universality and can easily achieve the delivery of various drugs. However, because there is no stable chemical bond between drugs and delivery microrobots in physical loading methods, drug leakage during drug delivery is often encountered. In contrast, chemical loading methods involve the formation of chemical bonds between drug molecules and delivery microrobots, resulting in greater difficulty in material selection for designing drug delivery microrobots. The correlation between delivery microrobots and drug types is enhanced, making it difficult to carry and deliver multiple drugs. The advantage of this delivery method is that the formed chemical bonds are usually more stable, reducing the risk of drug leakage during delivery.

After the drugs reach the vicinity of the lesion area, it is necessary for drug-loaded microrobots to accurately release the drugs. To achieve this, an understanding of the physiological environment near the lesion area is needed, especially some target signals that can serve as triggers for drug release, such as pH and enzymes. The key to drug release is that drug-loaded microrobots can smoothly release drugs when they reach the lesion area. Therefore, depending on the different loading methods, the drug release methods may vary slightly. For physical loading methods, the key is to reduce or release the physical confinement of drugs; while for chemical loading methods, drug release depends on breaking the chemical bonds between drug molecules and other molecules. In oral administration, the drug action site is generally the gastrointestinal tract. Therefore, differences in digestive enzymes, pH, geometric dimensions, and shapes in the gastrointestinal tract can be used as trigger conditions for drug release in the design of drug delivery microrobots. For example, Langer et al. utilized digestive enzymes in the stomach as a trigger condition [87]. In addition, drug release can also be achieved by the expansion or contraction of drug-loaded materials under specific conditions. A more universal method is to allow drug-loaded materials to degrade specifically near the lesion area. Clearly, drug loading and release methods should be closely related to specific oral therapeutic diseases and sites. Select appropriate target signals as triggers for drug release can improve the bioavailability and therapeutic effects of drugs.

### 4.2 Transportation

For oral drug delivery microrobots, transportation is crucial for achieving the therapeutic goals. In conventional oral drug therapy, drugs enter the human gastrointestinal tract through the mouth are generally transported within the human digestive system by gravity and peristalsis. This method is feasible for treatments where drugs only need to exert their effects in the stomach or intestines. For example, in oral insulin therapy or gastric retention systems, it is sufficient for the drug delivery microrobot to transport the drug to the gastrointestinal tract, and there is generally no significant demand for the mobility of such drug delivery microrobots [87]. Therefore, these drug delivery microrobots typically do not need to have active movement modules or only require minimal mobility.

Oral administration also includes precise targeted treatments for specific lesions in the gastrointestinal tract, which require drug delivery microrobots to have the ability to reach designated lesions, and even to reach some hard-to-reach areas within the gastrointestinal tract. To impart mobility to drug delivery robots, it is often necessary to add a locomotion module to them. The first consideration in designing locomotion modules is to choose an appropriate propulsion source. Currently, common propulsion methods in oral administration include magnetic drive and microbial drive, while light-drive, due to the limited penetration of visible light through deep tissues, is challenging to implement.

Magnetic field-driven locomotion modules typically use neodymium-iron-boron particles or ferrite particles of different sizes as magnetic drive materials. These magnetic particles are always doped into other materials to manufacture motion modules with specific structural shapes through 3D printing or molding. In some research, motion and deformation functions are designed simultaneously by continuously changing the magnetization direction of the magnetic particles during the manufacturing process [88]. Since neodymium-iron-boron magnetic particles are difficult to degrade directly in the body, attention should be paid to subsequent recovery and safety issues when using such magnetic drive designs.

Compared to magnetic field drive, microbial drive is a safer propulsion method. It involves attaching therapeutic drugs to microorganisms such as algae [20] or bacteria [24], utilizing their motility to achieve drug delivery in the gastrointestinal tract. Additionally, for self-propelled drug delivery microrobots, they have limited mobility and mainly rely on gravity and intestinal peristalsis to reach the site of drug action, generating propulsion through certain chemical reactions to drive their movement.

### 4.3 Overcoming the gastrointestinal barrier

In section 2, various barriers in the human gastrointestinal tract were introduced. While these barriers protect the safety of the human gastrointestinal tract, they also limit the efficacy of many drugs, especially biologics, in oral therapy. Therefore, it is crucial to overcome the gastrointestinal barriers in oral therapy.

The first challenge in oral drug therapy is to ensure the efficacy of drugs during the process of entering the body from the mouth and reaching the designated site of action, especially for drugs acting in the intestine, which must withstand the harsh and complex environment of the stomach after oral administration. A simple and effective method is to encapsulate the drug with appropriate capsules or drug delivery microrobots, which dissolve under specific conditions to release the drug [89]. For example, drugs intended to act in the stomach are encapsulated in gastric-resistant capsules, while those intended to act in the intestines are encapsulated in enteric-coated capsules.

Using capsules can only ensure that the drug safely reaches the vicinity of lesion site. However, different gastrointestinal barriers need to be overcome for different treatment modalities. One challenge is oral administration with biologic agents, where drug delivery microrobots need to overcome barriers such as proteases and mucosa in the gastrointestinal tract to deliver drugs into the human circulatory system for effective treatment. Traditional oral drugs use auxiliary additives such as protease inhibitors and permeation enhancers to protect drugs and improve their bioavailability [9]. While this approach is effective to some extent, the bioavailability of drugs remains relatively low. Some drug delivery microrobots use physical methods to pierce the drug delivery site into the gastric mucosa, and then directly release the drug below the mucosal layer, thereby greatly reducing the influence of mucosal and protease barriers [90]. For example, self-propelled drug delivery micro-robots pierce into the gastrointestinal wall through self-propulsion, after which the encapsulated drug layer dissolves, releasing the drug [91]. Some microneedle microrobots pierce into the mucosa through microneedles [92]. This method has higher controllability than self-propelled methods, but designing a driving module for accurate insertion of microneedles into the gastrointestinal wall is a challenge for microneedle drug delivery microrobots. Recently, rotating micro-robots in the stomach have shown significant improvements in drug therapy by continuously removing various barriers in the surrounding environment [93].

For some long-term continuous drug therapies in the gastrointestinal tract, drug delivery robots need to overcome continuous peristalsis and interference from food and other foreign substances in the body after eating. Using special structural designs such as micro-claws [94], microrobots can anchor in the gastrointestinal tract to resist peristalsis and interference from foreign substances. Moreover, research has found that using microalgae as oral drug carriers is beneficial for long-term sustained-release therapy [95]. For targeted drug therapy in the gastrointestinal tract or for drugs that are difficult to reach with conventional oral administration, the use of drug delivery microrobots with excellent mobility, such as some magnetic-controlled microrobots [96], can successfully reach areas that are difficult to access.

### 4.4 Fabrication

#### 4.4.1 3D printing

3D printing is a novel manufacturing method that converts 3D digital models created by Computer-Aided Design (CAD) into physical 3D objects layer by layer [97]. It offers advantages such as design freedom, high material utilization, ability to manufacture complex structures, high processing efficiency, and personalized customization [98]. In recent years, this advanced manufacturing method has rapidly developed in the pharmaceutical field [99]. Common 3D printing methods include Fused Deposition Modeling (FDM) [100], Stereolithography (SLA) [101], Digital Light Processing (DLP) [102], and more complex techniques like Two-Photon Polymerization (TPP) [103,104]. For the fabrication of drug delivery microrobots, different 3D printing methods can achieve rapid prototyping with printing precision ranging from 100 nm to several centimeters [101]. Additionally, the range of printable materials is continuously expanding, from synthetic materials like PLA to natural materials like proteins [105]. Some relatively complex structures, such as magnetic-driven drug delivery microrobots and anchored drug delivery microrobots, often involve special microstructure designs. Therefore, 3D printing is an effective solution for manufacturing such special structures. In the field of oral therapy, photopolymerization 3D printing is widely used due to its high printing precision and throughput [106]. This method manufactures high-resolution structures by polymerizing or fusing photosensitive materials. However, this printing method typically uses around 405 nm ultraviolet light, which can lead to drug denaturation upon exposure. Hence, it is not suitable for adding drugs during the printing process. Excitingly, a rapidly emerging volumetric 3D printing method also known as Computed Axial Lithography (CAL) has been developed, which is capable of shaping objects at different centimeter scales in 30–120 s [107]. This volumetric printing method utilizes longer wavelength green light as the light source, offering superior penetration and biocompatibility compared to traditional light-curing methods and has begun to demonstrate promising applications in biomedical fields [45]. It is believed that this low-cost, rapid-forming, non-contact printing method will also play a significant role in areas such as biopharmaceuticals and drug delivery microrobots in the future [108].

#### 4.4.2 Template-assisted electro-deposition

Template-assisted electro-deposition is commonly used in the manufacturing of self-propelled drug delivery robots that utilize metal layers generated by chemical reactions to produce bubble propulsion. This method can be used to add different coatings to the surface structure through electrodeposition. It relies on conically shaped micropores of the membrane template and involves sequential deposition of the outer polymeric layer and the inner catalytic metal layer [106]. Different electrodeposition conditions can achieve the manufacturing of multi-layered materials. For example, Gao et al. used this method to prepare PEDOT/Zn micromotors for drug delivery therapy in the stomach [109]. Since this method is generally used for the deposition manufacturing of nano-scale microstructures or for adding coatings to structures, it is difficult to manufacture large-scale structures. Therefore, template-assisted deposition methods are more commonly used for the functionalization of drug delivery robots in later stages.

#### 4.4.3 Molding

Molding primarily involves rapid manufacturing through mold casting and demolding processes using existing molds. Compared to 3D printing, this manufacturing method is simple, fast, suitable for mass production with minimal batch variations, and requires lower material requirements. For example, in anchored drug delivery microrobots, many of them utilize microneedles for drug loading design, which require relatively simple single-shaped molds and are easy to prepare [83,110]. Therefore, common microneedle structures are suitable for rapid manufacturing using molding. The mold manufacturing process requires consideration of the difficulty of mold manufacturing and the method of mold casting and demolding. Mold manufacturing can be achieved through 3D printing or traditional machining methods. As molding highly relies on molds, the cost of manufacturing complex molds is higher, and for some complex special structures, demolding processes have difficulties. Therefore, for personalized customization of some special complex structures, molding is not a good choice, while 3D printing methods are easier to implement and have much lower costs.

#### 4.4.4 Chemical conjugation

Chemical conjugation is commonly used in biohybrid drug delivery robots by connecting target materials with natural groups (such as -NH2) existing on the surface of microbial materials, thereby developing functionalized biohybrid drug delivery robots [79]. This coupling method can also load drugs onto the functionalized surface of other drug delivery microrobots.

In addition to using chemical conjugation, other methods such as self-assembly [111] and freeze-drying [20] are also commonly used effective methods for the manufacture or drug loading of biohybrid drug delivery microrobots.

## 5 Oral administration microrobots

A great amount of research has been conducted on oral administration microrobots in the past decades. In view of these research results, we will divide them into four parts according to the different driving methods: magnetic-controlled, anchored, self-propelled and biohybrid. Obviously, there are absolutely other microrobots driving by other methods, such as light, ultrasound and etc. But we would like to focus on the magnetic-controlled oral administration robots for magnetron is by far the most common, most studied and more reliable option.

Due to the difficulty in providing a comprehensive overview of the characteristics and functionalities of various oral drug delivery microrobots in this chapter, we have focused more on detailing the drug transportation methods and strategies for overcoming gastrointestinal barriers, which we believe are the key distinguishing features of microrobots from traditional therapeutic approaches. In addressing other key considerations mentioned in Chapter 4, we have created Table 2. In Table 2, we have extensively summarized the material composition, fabrication methods, loaded drugs, drug loading methods, applications, scale, as well as whether animal experiments have been conducted and the bioavailability of drugs for various types of oral drug delivery microrobots. We believe that by systematically presenting these crucial aspects, researchers in relevant fields can make intuitive comparisons, facilitating the design of more robust oral drug delivery microrobots.

**Table 2:** Schematic of the characteristic of different types of oral administration microrobots.

Propelled method	Name	Materials	Fabrication methods	Loaded drug	Drug loading Methods	Applications	Animal Experiment	Scale	Bioavailability	Ref
Magnetic	Magneto-Responsive Microneedle Robots	GelMA; BSA; PEGDA	Templated	Insulin	Microneedle	Diabetes therapy	Swine	About 3 mm in diameter and 1 mm in height	N/A	[83]
	Spinning-enabled wireless amphibious origami millirobot	Polypropylene film Ecoflex-0030 silicone; Hard-magnetic particles (NdFeB, average size of 100 μm); Glass bubbles	Assembling	N/A	Cavity	Cargo delivery	Swine	7.8 mm in diameter and 4.82 mm in height	N/A	[138]
	Millimeter-scale flexible robots	NdFeB; UV-resin (GC3D-EBE)	DLP-based lithography system; Mold	N/A	Microgripper	Cargo delivery	N/A	6mm × 6mm × 0.08 mm	N/A	[130]
	Small-scale soft-bodied robot with multimodal locomotion	Ecoflex 00–10 polymer matrix; NdFeB magnetic microparticles	Mold	N/A	Microgripper	Cargo delivery	N/A	3.7mm × 1.5mm × 0.185 mm	N/A	[88]
	Triple-Configurational Magnetic Robot	Genipin-induced chitosan; Sodium alginate (MW = 120–190 kDa), ${Fe}_{3}O_{4}$ MNPs	Pump the solution into 4 °C soybean oil to form uniform-sized GS; Electrodeposition	DOX	Form drug-loaded hydrogel	Targeted Drug Delivery and Sustained Release	N/A	Approximately 0.977 mm in diameter	N/A	[115]
	Reconfigurable Magnetic Slime Robot	NdFeB; Borax; Polyvinyl alcohol (PVA)	Mixing and stirring	N/A	Slime grasp	Transporting harmful things	N/A	N/A	N/A	[139]
	Wireless soft millirobots	Ecoflex 00–30 silicone rubber; NdFeB microparticles (average diameter, 5 μm); PDMS; P(NIPAm-co-AA) hydrogels	Mold; Photolithography; 3D printing	Fluorescence particles	pH-responsive hydrogels	Minimally invasive medicine applications.	N/A	About 3.7 mm in length, 1.5 mm in width, and 0.15 mm in height	N/A	[124]
	Multi-legged soft millirobot	Polydimethylsiloxane (PDMS); Iron microparticles; Polystyrene plate	Modified magnetic particle assisted molding approach	N/A	Tablet	In vivo medical transportation	N/A	Length of 17 mm, the width of 7 mm and the thickness of 150 μm	N/A	[125]
Anchor	Self-orienting millimeter-scale applicator (SOMA)	PEO (MW: 200 k); PCL; 316 L stainless steel	3D Printing; Mold	Insulin	Compress insulin and PEO into millipost	Diabetes therapy and other macromolecules oral delivery	Swine	Smaller than φ9mmX15mm	approximating subcutaneous administration bioavailability.	[87]
	The liquid-injecting self-orienting millimeter-scale applicator (L-SOMA)	VisiJet SL Flex ink (3D Systems); 304 stainless steels; Polyoxymethylene; Polyphenylene ether; Isomalt	3D Printing injection-molded	Adalimumab, insulin, GLP-1; Epinephrine	Using a hypodermic needle to load liquid drug formulation	Diabetes therapy and other macromolecules oral delivery	Swine	12 mm in diameter and 15 mm in height	80 %	[90]
	Luminal unfolding microneedle injector (LUMI)	Poly (methacrylic acid-*co*-ethyl acrylate) coating; Polyethylene glycol (PEG) (molecular weight: 3500) coating; Polyethylene oxide (PEO); Soluplus (polyvinyl caprolactam-polyvinyl acetate-polyethylene glycol graft copolymer)	3D Printing; Mold	Recombinant human insulin	Drug-loaded microneedles	Diabetes therapy and other macromolecules oral delivery	Swine	9 mm in diameter and 30 mm in length	>10 %	[92]
	A millisecond self-oriented microneedle containing robots	Resin (OBJ-04057); PVA/PVP; Chitosan	Stereolithography	N/A	Drug-loaded microneedles	Chronic inflammation and cancer of the colon	Zebra swine	14 mm in diameter and 12 mm in height	N/A	[192]
	Robotic mucus-clearing capsule (RoboCap)	VeroClear photopolymer; Gelatin; Eudragit-L	3D printing (Stratasys)	Vancomycin; Insulin	Using a compartment house to load drug	Vancomycin and Insulin oral delivery	Yorkshire swine	Sized as a triple-zero capsule	20- to 40-fold greater than standard oral delivery	[93]
	Dynamic omnidirectional adhesive microneedle system (DOAMS)	VeroClear resin; Flexibale resin; isomalt; PCL (MW: 45 kDa)	Mold; 3D printing	Semaglutide with SNAC	Drug-loaded microneedles	Diabetes therapy and other macromolecules oral delivery	Yorkshire swine	N/A	N/A	[110]
	Suction cup orifice design (SCOD)	Poly (β-thioether ester) polymer resist	Digital Light Processing	Desmopressin; Semaglutide	The suction patches	Boosting systemic absorption of peptides	Beagle dogs	11 mm in diameter and 6 mm in height	16.4 %	[85]
	A single-encounter oral, ultra–long-acting form of ivermectin	Formulations containing ivermectin; excipient polymers such as pluronic P407, and linear PCL polymer; PCL-based PU thermoset elastomer; Eudragit L100-55 (Evonik)	Mold	Ivermectin	Drug-loaded polymer	Malaria	Yorkshire swine	Cross-sectional dimension of less than 2 cm	N/A	[168]
	Esophageal and gastric-resident drug delivery systems	A thermoplastic polyester; PCL (MW, 40 kDa); Elastollan 1185 A (BASF); Shape-memory nitinol (NiTi)	Mold	Budesonide	Drug-loaded millineedle	Controlled drug delivery systems in the GI tract	Yorkshire swine	About 6.8mm × 6.8mm × 15mm	N/A	[170]
	Soft ingestible hydrogels	Polyvinyl alcohol powders (PVA; MW 146000–186,000); Radio-opaque barium sulfate; Superabsorbent particles (sodium polyacrylate homopolymers); Ethyl cyanoacrylate glue	Laser cutting; Mold; Assemble	N/A	N/A	Long-term gastric retention and physiological monitoring	Yorkshire swine	Diameter of 1 cm and length of 3 cm	N/A	[159]
	Gastrointestinal-resident, shape-changing microdevices	Copper; Silicon wafer; Cr; Au; S1813 photoresist; SPPR220 photoresist; Chitosan; AZ9260 photoresist; Paraffin wax	Thermal evaporation; Thin-film evaporation; Electrodeposition; Photolithography	Ketorolac	Controlled drug release matrix	Extended-release drug delivery	Mice	250 μm size	N/A	[94]
	An intestinal peristalsis–actuated microneedle robot	PEGDA/PEG; PVA/Aam; High-viscosity sodium carboxymethyl cellulose	Template-assisted photo-crosslinking method	Insulin	Drug-loaded microneedles	Diabetes therapy and other macromolecules oral delivery	Bama minipigs	Smaller than a commercial 00# capsule	23.6 %	[165]
self-propelled	Rocket-inspired effervescent motors (RIEMs)	GelMA; HMPP; PVP; Effervescent powder	Mold	Insulin	Drug-loaded microneedle tips	Diabetes therapy and other macromolecules oral delivery	Rabbit	N/A	N/A	[89]
	Poly (aspartic acid)-based micro-rockets	Zn; Fe; Poly (aspartic acid) (PASP)	Electrodeposition	DOX	Electrostatic interaction	Targeted delivery of an anticancer drug in vivo.	ICR male mice	N/A	N/A	[91]
	Biomimetic Micromotor Enables Active Delivery of Antigens	Mg; TiO2; Chitosan Enteric coating (Eudragit L100-55); Red blood cell (RBC) membrane	Atomic layer deposition and solvent evaporation process.	Motor toxoids	Coated with the toxin-bound RBC membrane	Oral antivirulence vaccine	Male CD-1 mice (Envigo)	An average size of 20 ± 5 μm in diameter	N/A	[179]
	A drug-loaded Mg-based micromotor	Magnesium (Mg) microparticles; TiO2; Drug@PLGA; Chitosan	Electrodeposition	Clarithromycin	coated with a PLGA film containing drug payload	Stomach infection therapy	Mice	An average size of ∼20 μm.	N/A	[177]
	Multicompartment tubular micromotors	An enteric coating film (Eudragit L100); PEDOT; Au; Zn; Rh6G-loaded gelatin	Electro-polymerization; Electrodeposition	Rh6G dye as model drug	Cavity	Localized Active Delivery	Mice	≈5 μm diameter and ≈15 μm length	N/A	[182]
	Metformin microstirring pills	Mg microparticles; TiO2; Lactose; Maltose; Metformin hydrochloride; Eudragit L-100	Atomic layer deposition; Coating technique	Metformin hydrochloride	Pill	Type 2 diabetes mellitus therapy	Male CD-1 mice	Diameter of 2 mm and length of 3 mm	N/A	[183]
Biohybrid	Oral drug delivery system, SP@AMF	Spirulina platensis	Chemical synthesis	Amifostine (AMF)	Lyophilization	Intestinal protection in cancer radiotherapy	Mice	Micron size	N/A	[20]
	An extremophile-based biohybrid micromotor	C. pitschmannii CPCC 354	Chemical	Poly (lactic-*co*-glycolic acid) (PLGA) nanoparticles	A bioorthogonal conjugation approach using copper-free click chemistry	Biomedical operations in harsh acidic environments	Mice	N/A	N/A	[191]
	Microalga-based multifunctional oral, cross-linking hydrogel BBR-CV@ALG	Chlorella vulgaris (CV), Berberine (BBR), carboxymethyl chitosan/sodium alginate complex	Chemical crosslinking	Anti-inflammation drug molecules	Form multifunctional hydrogel	Lead poisoning treatment	Mice	N/A	N/A	[17]
	An efficient algae-based motor platform	Chlamydomonas reinhardtii, Enteric-coated capsule	Chemical Conjugation	DOX	Conjugation by click chemistry	Enhance algae motors utility for GI delivery applications	Mice	N/A	N/A	[95]
	An orally used microalgae-nano integrated system (SP@ASXnano)	Spirulina platensis (SP), poly (lactic-*co*-glycolic acid) (PLGA), chitosan	Chemical Synthesis	Astaxanthin	Electrostatic adsorption	Against radiation-induced injury	Mice	N/A	N/A	[16]
	Twin-bioengine self-adaptive micro/nanorobots	Baker's yeast (*S. cerevisiae*), Glucose oxidase, Catalase	NP self-deposition, asymmetric surface activation, and GOx/Cat immobilization	Curcumin	Conjugation	GI inflammation therapy	Mice	N/A	N/A	[79]
	A microalgae-based oral drug delivery system (SP@Curcumin)	Spirulina platensis (SP)	Chemical Conjugation	Curcumin	Soak	Colon cancer and colitis	Mice	100 μm long and 5 μm width	N/A	[190]

### 5.1 Magnetic-controlled drug delivery microrobots

#### 5.1.1 Rigid magnetic-controlled microrobots

For oral administration, the complicated gastrointestinal environment is a barrier to achieve flexible movement for drug delivery microrobots. As the magnetic actuation which can be driven wirelessly by an external magnetic field, has the capability of providing necessary power and precise control of the microrobot [112], delivering, and releasing drug in gastrointestinal tract have become much easier. Magnetic-controlled drug delivery microrobots have shown a wide range of applications in biomedical fields, especially in oral administration.

**Fig. 2:**
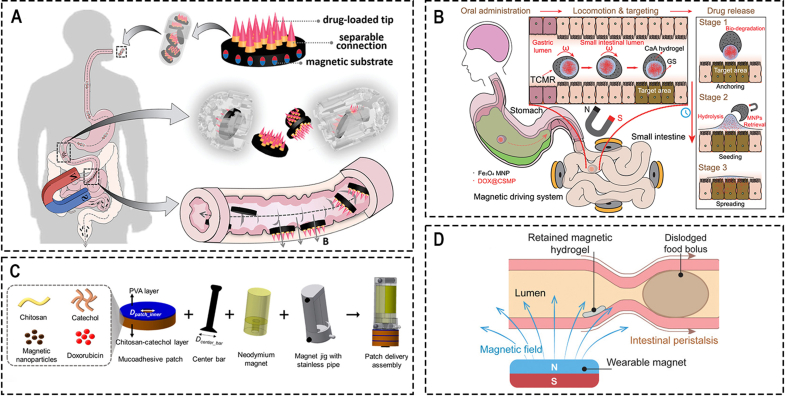
Conventional Magnetic-controlled Microrobots. (A) Oral magneto-responsive microneedle robots for intestinal macromolecule delivery [83]. ***Copyright ©2021 Wiley-VCH GmbH***; (B) Untethered triple-configurational magnetic robot for intestinal targeted drug delivery and sustained release [115]. ***Copyright ©2021 American Chemical Society***; (C) A magnetically actuated capsule with multi-layer drug-loaded microneedle patches [114]. ***Copyright ©2022 Wiley-VCH GmbH***; (D) An ingestible magnetic hydrogel carrier [119]. ***Copyright ©2021 Wiley-VCH GmbH***.

Zhang et al. [83] presented a novel magneto-responsive microneedle robot, which consists of three components of magnetic substrate, the separable connection, and tips (Fig. 2A). This robot can be wrapped in a commercial enteric capsule, so it can be taken orally and released in the intestine. Due to the magnetic substrate, the robot can move under the action of a permanent magnet and inserts microneedles into the intestinal mucosa to release the loaded drug, after the separable connection is degraded, the magnetic substrate will be excreted outside of the body with the help of the external magnet. Based on the same principle, but using a rotating magnetic field to realize more precise control, Lee et al. [113] proposed a magnetically actuated capsule with multi-layer drug-loaded microneedle patches. This capsule can change its positions between the locomotion state and the delivery state under the control of an external rotating magnetic field. Once the capsule is driven to the desired target lesions, it can move to a vertical position and insert the drug-loaded microneedle (MN) patch into the lesions. Additionally, they also developed a magnetically actuated capsule with multi-layer drug-loaded mucoadhesive patch for gastrointestinal tracts (Fig. 2C) [114].

For those robots with drug-loaded microneedles, a magnetic field is just used to control the movement of the microrobots, drug release process occurs through the degradation process of the microneedle. Though, this method is essential in some conditions, such as delivery insulin, for other drugs that can release directly in the gastrointestinal tract, this method has low efficiency and a little bit complicated. Therefore, Zhou et al. [117] proposed a novel magnetically actuated robotic capsule for site-specific drug delivery inside the gastrointestinal tract. This robotic capsule consists of two hemispherical bodies, each hemispherical body has two magnetic membranes and one needle. Magnetic membranes are used to wirelessly provide magnetic actuation for releasing the drug and controlling the protrusion and retraction of the needle respectively. By changing the intensity of the external magnetic field, the amount of the deflection can be adjusted, thus, the released amount of payload can be controlled. In this study, both locomotion and drug injection tests were successfully performed in vitro experiments. Hua et al. [116] proposed a ferrofluid soft capsule robot for diagnostic and drug releasing inside the human stomach, which equipped with a composite soft shell and a specific oscillation module to make the locomotion more controllable and secure. Also, Wang et al. [118] realized the sustained-release and quantitative drug delivery based on the characteristics of mini-magnets’ individual starting frequency- and pitch-related calculations. And this can be used to miniaturize the independent drug delivery module.

Though, above mentioned drug delivery microrobots have shown great potential in oral administration, and targeted therapies, these microrobots still confront challenges, such as biocompatibility, sustained-release capabilities and biodegradability. Hydrogels, as a biocompatible material have widely used in biomedical fields, thus, develop drug delivery robot with hydrogels have the potential to solve this problem. Chen et al. [115] developed an untethered triple-configurational magnetic robot (TCMR) for bowel cancer-targeted therapy (Fig. 2B). This robot consists of three parts: actuation and guarding, anchoring and seeding, and drug release part. Its shell, which acts as the actuation and guarding part that contains magnetic nanoparticles (MNPs) and cargo, can degrade in the intestinal juice (pH = 7.4). Once the shell is degraded in the intestinal, the robot will be separated to two parts: a gelatin sphere, which serve as the anchoring and seeding part to release the drug slowly through a hydrolysis process, and the shell encapsulates the vast majority of MNPs will be retrieval after drug delivery using an external magnetic field. To address the localization and sustained drug delivery of magnetic-controlled microrobot, Liu et al. [119] presented an ingestible magnetic hydrogel carrier for transporting diagnostic microbes to specific intestinal sites and prolonging monitoring and modulation of the digestive system and adjacent organs (Fig. 2D).

#### 5.1.2 Untethered Magnetic-controlled soft microrobots

Untethered magnetic-controlled soft microrobots, with their small size, continuous deformation, infinite degrees of freedom, and the compliance and mechanical properties make them especially popular for medical applications [[120], [121], [122]], such as drug delivery [123], and targeted therapy. However, soft robots need corresponding actuators to provide enough power to achieve movement. Since magnetic actuators are able to be driven wirelessly and controlled flexible by a magnetic field, exploiting the concept of magnetism to generate force and torque for novel medical robots has been considered and proven as a promising technique [112]. Combining the untethered small-scale soft robots and magnetic actuators gives robots strong locomotion abilities and enable to non-invasively access to narrow and complex space. Thus, these magnetic-controlled soft microrobots potentially have a wide range of applications in biomedical therapeutics.

**Fig. 3:**
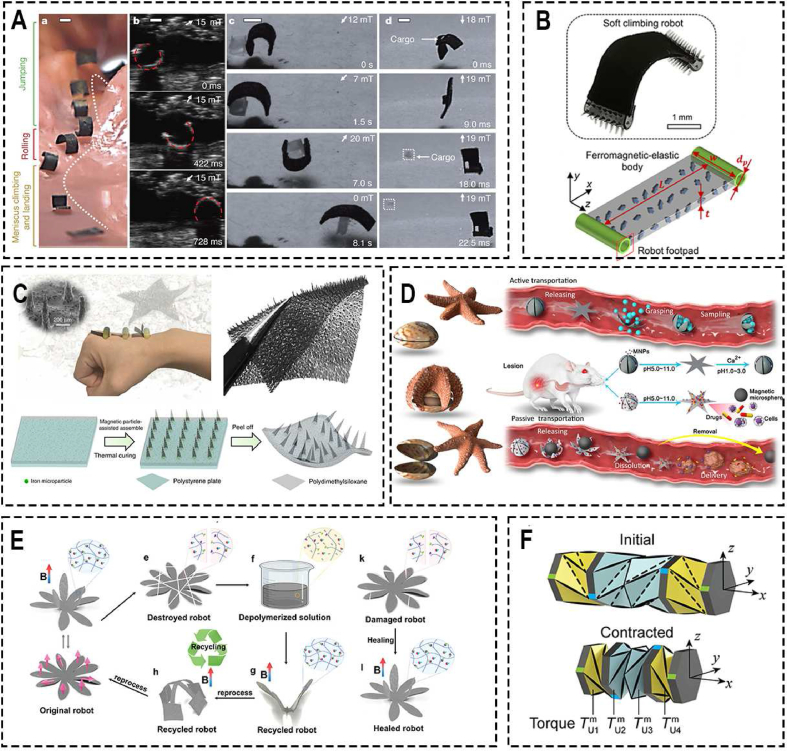
Untethered Magnetic-controlled Microrobots. (A)Magneto-elastic soft millimeter-scale robot with multimodal locomotion within narrow tunnels [88]**. *Copyright ©2018 Spinger Nature***; (B)Wireless soft millirobots based on a fundamental peeling-and-loading mechanism to achieve climbing three-dimensional dry and wet surface in confined spaces [124]**. *Copyright ©2022 AAAS***; (C) elastic-leg-inspired untethered soft millirobot which demonstrate many superior functionalities in both wet and dry conditions [125]. **. Copyright *Copyright ©2018 Spinger Nature***; (D)Starfish inspired programmable ionic shape-morphing micro-robotic end-effectors for environmentally adaptive targeting, releasing, and sampling [126]**. Copyright *©2021 Spinger Nature***; (E)A radio frequency identification based battery-less soft millirobot with multilayers structure, which can achieve multi-functionalities in the gastrointestinal tract [127]***. Copyright ©2020 Wiley-VCH GmbH***; (F)Fully recyclable, healable, soft, and stretchable dynamic polymers for magnetic soft robots [128] ***Copyright ©2023 Wiley-VCH GmbH***.

As rigid microrobots have limited mobility, most of them can only perform one single motion, such as crawling, or swimming, which limits their applications in complex biomedical microenvironments. So soft microrobots with multimodal locomotion, precise control, flexible deformation are desired by researchers, and have the potential applications in oral drug delivery. To achieve these multi-functional soft microrobots, one possible way is to use the principle of liquid stratification and interfacial diffusion [129], more popular way is to insert the magnetic nanoparticles into the elastic materials or hydrogels and conduct magnetization programming. Hu et al. [88] designed a magneto-elastic soft robot which can be programmed to have a single-wavelength harmonic magnetization profile M along its body, and then controlled by a time-varying magnetic field B to generate different modes of locomotion, including swim inside and on the surface of liquids, climb liquid menisci, roll and walk on solid surfaces, jump over obstacles, and crawl within narrow tunnels (Fig. 3A). With these locomotive modes, it can execute pick-and-place, cargo delivery and cargo-release tasks. Different from Hu's idea, Xu et al. [130] proposed an ultraviolet (UV) lithography-based method to encode 3D magnetization in planar flexible composites at the submillimeter scale. Based on this method, they fabricated millimeter-scale microrobot that exhibited higher-order and multi-axis deformation, large-angle bending, or combine bending and torsion. In their study, they also have shown this robot's ability of cargo delivery in vitro. Zhao et al. [131] realized multi-modal motion of the soft microrobot by using a heating-free reprogrammable magnetization technique to regulate the robot's response actions through re-patterning the magnetic domain, additionally, they also employed a stiffness programming technique in terms of the liquid-metal thermotropic phase transition to regulate the robot's stiffness. As magnetic hydrogels have demonstrated great potential in soft robot and drug delivery for their biocompatibility and biodegradability, but the magnetic domains of the magnetic hydrogels embedded with soft magnetic particles cannot be programmed and retained under external magnetic fields. Thus Tang et al. [132] used direct ink writing to extrude the magnetic hydrogels precursor to fabricate 3D structures with fast actuation. Goudu et al. [133] achieved 3D magnetization programming inside the hydrogel body by directionally self-assembled superparamagnetic iron oxide nanoparticles (SPIONs) chains using an external permanent magnet. Based on this strategy, they developed a hydrogel milli-gripper that can perform cargo grabbing and delivery and can be degraded completely. By programming the electric field, Zheng et al. [126,134] proposed a single-step aniso-electrodeposition method for fabricating modular microrobots (MMRs) with distinct functions in each modular segment and can be endowed with diverse shape-morphing capabilities (Fig. 3D). Based on this method, combined with the ionic sensitivity of alginate hydrogel microstructure, they presented a shape-morphing strategy for microrobotic end-effectors to adapt to different physiochemical environments and accomplish tasks such as targeting, releasing and sampling under the control of a magnetic field and environmental ionic stimuli. The versatility was demonstrated experimentally in both in vitro environments and ex vivo in a gastrointestinal tract. One main limitation for existing microrobots is how to integrate multiple functional modules with dissimilar material compositions into one soft robot. To overcome this limitation and to create magnetic soft micro-robots with programmable magnetization profiles (uniform or non-uniform), multi-modal motion, and 2D or 3D geometries of various functions, a single modular soft magnetic unit containing NdFeB particles with prescribe magnetization profiles which is directly embedded in the adhesive layer network was developed [135]. The authors also demonstrate the potential of the soft microrobot in treating gastric ulcers with the assistance of an ultra-fine wired endoscope Table 3.

**Table 3:** Advantages, limitations, treatable diseases and clinical translation challenges of microrobots.

Type of Microrobots	Advantages	Limitations	Typical treatment disease	Clinical translation challenge
Magnetic-controlled drug delivery microrobot	High controllability and mobility.	Poor biocompatibility and biodegradability	Treatment of gastrointestinal tumors	Lack of corresponding in vivo animal studies on oral drug delivery
	Capable of specific deformation and precise motion.	Treatment process heavily reliant on magnetic driving equipment	Targeted drug therapy at the bile duct	Compatibility issues between magnetic -driving equipment and hospital equipment, as well as safety concerns for patients and clinicians
	Ability to achieve precise targeted therapy.		Diabetes	
Anchored drug delivery microrobot	High bioavailability of drugs	Lack of autonomous mobility, low targeting capability	Insulin	Preclinical large animal and primate repeat dosing experiments.
	High drug payload	Large size, risk of gastrointestinal obstruction	Tuberculosis	Long-term repeat dosing experiments in clinical settings
	Simple operation and low cost		Malaria	
	Prolonged drug therapy duration			
Self-propelled drug delivery microrobot	Limited active mobility, able to overcome gastrointestinal barriers.	Short propulsion lifespan	Insulin	Drug delivery studies in large animal models.
	Small structural size, less prone to intestinal obstruction	Autonomous motion process less controllable, low efficiency	Gastric ulcer	Improvement of propulsion lifespan and enhancement of drug delivery robot biocompatibility
	Self-powered, leaving minimal residue.	Low targeting capability	Ulcerative colitis	
Biohybrid drug delivery microrobot	Better biocompatibility and biodegradability	Poor controllability	Oral treatment of metal poisoning intestinal radioprotection	Drug delivery studies in large animal models.
	Can sustain continuous propulsion without relying on external fields, with long propulsion lifespan.	Some bacterial drug delivery robots pose risks of pathogenicity and immunogenicity.	Colorectal cancer and colitis	Selection of appropriate microbial carriers for corresponding disease types, optimization of drug molecule loading processes.
	Able to sense changes in the surrounding environment.			Research on risks such as microbial pathogenicity or immunogenicity
	Wide range of raw material source			

Different from those robots achieved multimodal locomotion by programming magnetic microparticles. Wu et al. [124] developed a wireless soft climbing robot by using different adhesive footpad designs that integrates both microstructures and tough bio-adhesives, which can achieve controllable adhesion and friction to locomote on various wet and soft tissue surface (Fig. 3B). Inspired by the wide range of function of the animals’ legs/foots, Lu et al. [125] reported an untethered soft millirobot decorated with tapered feet structures (Fig. 3C). Moreover, they have integrated the power generation and actuation functions in a multilayer thin film (<0.5 mm) for the first time, and thus reported a battery-less soft millirobot [127], which represents a remarkable advance in the emerging area of untethered soft robotics. Beyond realizing multimodal locomotion, adaptability and self-healing abilities are also crucial for soft robots. Zhu et al. [128] designed a dynamic covalent polyimide as polymer matrix and magnetic NdFeB particles as filler based on the synergistic effect of reduce cross-linking density by Schiff base reactions and reduced intermolecular hydrogen bonding, realizing a single magnetic soft robot with complete chemical recoverability, room temperature self-healing ability and multi-mode actuation set integration (Fig. 3E). The robots can be used for a variety of future applications, going beyond the limited and fixed inherent geometry of traditional soft robots.

Another kind of soft microrobot is the origami microrobot. Origami technology as a novel technology, with the properties of scalability, tunability of geometry, reconfigurability and easiness in manufacturing, it has been adopted to explore novel solutions to existing problems across different fields [136]. Especially, with the capability of origami to move from a folded to a deployed state, the origami robot has attracted great attention in the biomedical fields. Ze et al. [137,138] creatively reported a magnetically actuated amphibious origami millirobot based on the triangulated cylindrical Kresling origami (Fig. 3F). The robot has the ability of self-adaptive locomotion, which can navigate on different terrains by overcoming various obstacles through a self-selected locomotion mode based on the surface feature, including rolling, flipping, and swimming. Due to its foldable thin-shell structure, geometrical features, and external three-dimensional magnetic field, the origami millirobot can achieve liquid medicine delivery, and cargo transportation. The experiments in ex vivo animal organs demonstrate this robot can be applicated in complex biomedical environments, like gastrointestinal tract. The author anticipates the multi-functional magnetic amphibious origami millirobots could potentially serve as minimally invasive devices for biomedical diagnoses and treatments. As a lot of soft robots are fabricated by elastomers, this type of material has limited deformability, which makes elastomer-based small-scale soft robot cannot navigate through highly restricted environments. Based on liquid metal have better deformability and flow properties, small fluid-based robots have been developed to pass through extraordinarily narrow and restricted space and avoid damaging surrounding biological tissues. So, Sun et al. developed non-Newtonian fluid-based magnetically actuated miniature soft robots to implement multiple functions [139]. Furthermore, Sun et al. [140] exploited the wetting properties and reconfigurability of ferrofluids to construct multifunctional miniature soft machines, which can adapt to changing terrain in unstructured environments. A single droplet can be controlled to split into multiple sub-droplets and can be re-fused as needed. Ferrofluid droplets can be configured as liquid capsules, enabling drug delivery therapy in the gastrointestinal tract. Though these small-scale soft robots have demonstrated great abilities of movement, control precision and drug delivery in harsh environments. Also, these robots show large potential in oral administration. However, there are seldom studies using these soft robots to load and release drugs in vivo. Expected in the future, there will be more animal experiments in vivo to show the efficacy of these soft robots on loading and releasing drugs.

#### 5.1.3 The intelligent magnet-controlled microrobots and swarms

Although the targeted delivery of microrobots to multiple organs and large chambers, such as gastrointestinal tract, has been realized, there are still some deep and narrow spaces in the human digestive system that are difficult for conventional oral administration to access directly, including tiny chambers and curved conduits located on the circulatory system and bile ducts. To make oral administration of these hard-to-reach regions accessible, in contrast to the mentioned two types of magnetic-controlled drug delivery microrobots, smaller-scale sub-millimeter magnetic drug delivery microrobots have also begun to attract researchers' attention. These microrobots can achieve the highest precise control, and due to their particularly small size, they can reach some hard-to-reach areas in the gastrointestinal tract. Combined with advanced control strategies and other sophisticated medical devices, these magnetic drug delivery microrobots can exhibit higher intelligence. They are better able to adapt to high-precision oral targeted drug delivery treatments that are difficult to achieve using conventional methods.

Inspired by bacterial flagella, Zhang et al. [141] reported a microscopic artificial swimmers by using helical propulsion for the first time. The experiments had demonstrated that this robot can propelled and steered precisely in water by a low strength, rotating magnetic field. This study has propelled the development of such micro-helical robot. Park et al. [142] reported a degradable hyperthermia microrobot (DHM) with a 3D helical structure, which remotely and precisely controlled to a target area by an external rotating magnetic field (RMF). The DHMs are fabricated by TPP using a PEGDA-PETA-based stealth polymer that contains Fe_3_O_4_ magnetic nanoparticles (MNPs) and 5-fluorouracil (5-FU). This robot can elevate the temperature under an alternating magnetic field (AMF), so 5-FU can be released from the DHMs in normal, high-burst, and constant release modes to control drug release and reduce the viability of cancer cells. The author expected these biodegradable DHMs to propel the development of actively controlled drug release and hyperthermia therapy. Also based on this kind of micro-helical robot, Lin et al. [143] proposed a magnetic driven double curved conical micro-helical robot (DCCMHR) by optimizing the geometric structure and the geometric parameters of the helical. The swimming speed and stability of this novel micro-helical robot has been greatly improved. Terzopoulou et al. [144] by combining micro-helical robots and metal-organic frameworks (MOFs), had developed a MOF-based small-scale robots (MOFBOTs). The main feature of this robot is that it can fully degrade in the cell culture condition.

As mentioned above, micro-helical drug delivery robots are capable of non-invasive, flexible movement under the control of external magnetic field, and small size. Also, they can be fabricated with degradable materials. Due to these characteristics, micro-helical drug delivery robots demonstrate the potential of biomedical therapeutic. However, this kind of robot exists an inherent problem that the microrobot must contain large amount of magnetic nanoparticles (MNPs) due to magnetic actuation. If a large number of MNPs remain in the patients’ body, they may affect cell metabolism and cause side effects. Based on this issue, Lee et al. [145] using disulfide bond, which can be separated through the external stimulus near-infrared (NIR) and dithiothreitol (DTT), to attach the MNPs to the fabricated helical microrobot. So, they reported a biocompatible and hydrolysable PEGDA-based drug delivery helical microrobot capable of MNPs retrieval. MNPs were retrieved through electromagnetic fields. *In vitro* validation proved that the proposed microrobot is suitable for cancer cell treatment.

Though a single microrobot can achieve high precise control, it has many shortcomings due to its small size, a single microrobot has many shortcomings due to its small size, such as small drug load, unable to track in vivo, large environmental interference factors, limited functions and so on. As a result, researchers have paid attention to the microrobot swarms, and navigating large swarms of micro/nano-robots is critical for targeted delivery/therapeutic applications. Yang et al. [146] proposed a framework that defines different levels of autonomy for crowd navigation of an environmentally adaptive microrobot and designs corresponding system components for each level by using deep learning algorithms. The intelligent decision-making behavior of micro/nanorobots with highly autonomous distribution planning in real time according to environmental changes is realized. This technology makes the targeted treatment of micro/nano-robot swarms more controllable and precise, whether in the gastrointestinal tract or in the complex blood circulation system of the human body. Based on micro/nano-robot swarms, Wang et al. [147,148] provide a detailed review of recent advances in swarm behavior driven by external dynamics, including basic understanding of swarm formation, navigation, and mode transformation. Except micro/nano-robots swarms, flexible guidewire/catheter have also been developed to access complex and hard-to-reach regions. Yang et al. [149] integrated the merits of both wired and wireless microrobots to design a novel wired magnetic microrobot (WMM), which can realize simultaneous multimode magnetic control and US(ultrasound) tracking of the microrobot in a complex tissue-mimicking environment.

To delivery microrobots quickly and with high precision into the bile duct, Wang et al. [150] combined tethered endoscope and non-tethered soft microrobots, giving full play to the advantages of both, and developed a soft and resilient magnetic stem cell spheroid microrobot (MSCSMs) with high biocompatibility. Using the three-dimensional (3D) self-assembly of stem cells, doping low-dose magnetic particles. An integrated robotic platform called Endoscopy-assisted Magnetic Actuation with a Dual Imaging System (EMADIS) has been developed for precise motion control of the magnetic microrobot to the target position after it is quickly delivered to the bile duct through the endoscope. EMADIS delivers MSCSMs precisely into the bile duct through the esophagus, a total distance of about 100 cm, within just 8 min, and has demonstrated excellent therapeutic results. This combination of endoscopes and microrobots opens a promising therapeutic approach with highly extended working distances, improved time efficiency for remote delivery, and multiple functions with high clinical value.

**Fig. 4:**
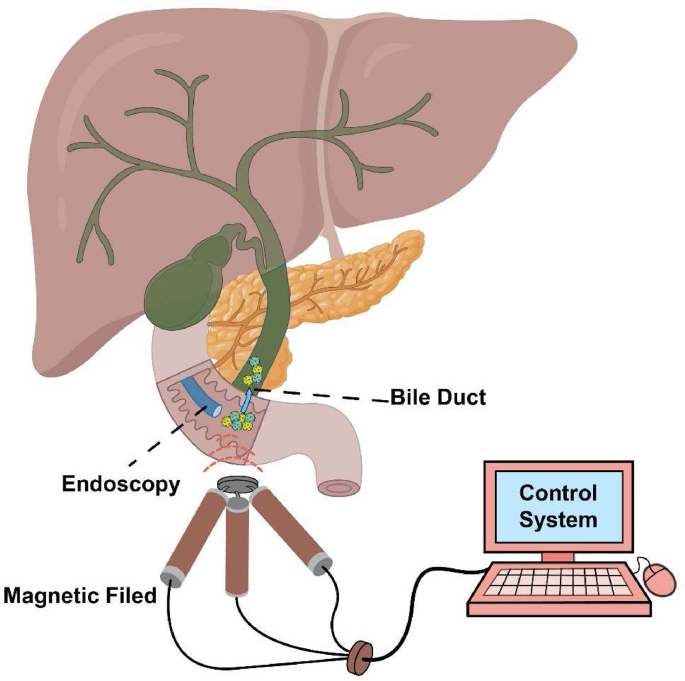
Combination of computer-assisted controlled endoscopy and magnetic field for hepatobiliary and pancreatic partial drug delivery.

Both microrobot swarms and catheters provide possibilities for targeted therapies in challenging areas of the human body, particularly in the bile duct, liver, hepatobiliary and pancreatic parts of the digestive system. Conventional oral administration struggles to deliver drugs to these regions. Utilizing methods such as endoscope-assisted micro/nanorobot swarms enables rapid, highly precise targeted transport, offering a novel and effective approach for oral treatments of hepatobiliary and pancreatic parts diseases, as shown in Fig. 4. Therefore, for oral treatments targeting specific regions and to address the complex internal environment, integrating advanced control systems, medical imaging devices, and automated control algorithms to enhance the efficiency of targeted delivery for microrobots is a promising avenue. For future development, two important questions need to be answered. One is how many microrobots are necessary to deliver a therapeutic dose of a drug to a target tissue? And the other is how will a medical procedure be performed with these devices [151].

### 5.2 Anchored drug delivery microrobots

As for the magnetic-controlled drug delivery microrobot which is movable introduced above, they can achieve various complex locomotion in the body under the action of external alternating magnetic field. This is very promising for drugs that need targeted therapy or the drugs that need to be precisely located to the target lesions and release. However, for some common macromolecule peptide and protein drug therapy or for some chronic diseases that need frequent administration, such as diabetes. Facing these conditions, magnetic-controlled microrobots may not be a suitable choice. On the one hand, the use of magnetic-controlled microrobots for drug delivery increases the complexity of the treatment; on the other hand, the influence of the human body constantly in the alternating magnetic field also needs further investigated. Therefore, subcutaneous injection is still the standard mode of administration of these drugs. Whereas long-term subcutaneous injection of drugs therapeutic has brought great physical and psychological trauma to patients [152,153]. Though various transdermal [154,155] and oral cavity [156] drug delivery microrobots have been developed, still, oral delivery of these macromolecule peptide and protein drug faces substantial barriers related to the structural organization and physiological function of the gastrointestinal tract [157]. Gastrointestinal epithelium acts as a physical and biochemical barrier for absorption of proteins resulting in low bioavailability (typically less than 1–2%) [2], people still prefer oral administration due to its high patients compliance, low cost, and easy to use [158]. Fortunately, by designing ingenious mechanical devices with microneedles loaded with drugs and using the natural conditions of the human gastrointestinal tract as triggers, such as pH, researchers have been able to develop some oral macromolecule drug delivery devices that allow insulin to be released into the gastrointestinal mucosa.

**Fig. 5:**
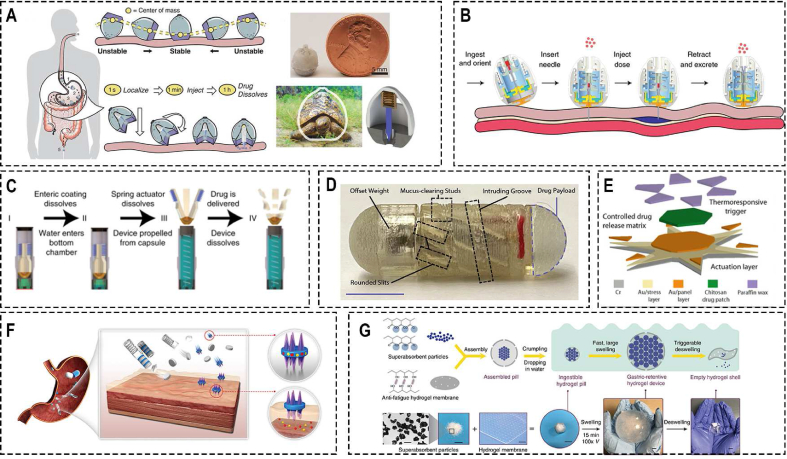
Anchored Drug Delivery Microrobots. (A)An ingestible self-orienting system for oral delivery of macromolecules [87]***. Copyright ©2019 AAAS***; (B)An orally dosed capsule for gastric injections of liquid formulations [90]***. Copyright ©2021 Springer Nature***; (C)An ingestible unfolding microneedle injector, which can propel dissolvable drug-loaded microneedles into intestinal tissue [92]***. Copyright ©2019 Springer Nature***; (D) An orally robotic drug delivery capsule that locally clears the mucus layer to enhanced drug delivery in the gastrointestinal tract [93]***. Copyright ©2022 AAAS***; (E) GI parasite-inspired active mechanochemical therapeutic grippers with the ability to autonomously latch onto the mucosal tissue to prolong reside time [94]***. Copyright ©2020 AAAS***; (F)The dynamic omnidirectional adhesive microneedle system (DOAMS) [110]***. Copyright ©2022 AAAS***; (G) The fabrication process of the pufferfish-inspired ingestible hydrogel device [159]***. Copyright ©2019 Springer Nature***.

Inspired by the leopard tortoise's ability to passively reorient, Abramson et al. [87] developed an ingestible self-orienting millimeter-scale applicator (SOMA) (Fig. 5A). This applicator can autonomously position itself to engage with gastrointestinal (GI) tissue. The authors also conducted in vivo studies in rats and swine to confirm the safety of this applicator. They believed the SOMA provides a way to deliver insulin orally and could potentially be used to administer other active pharmaceutical ingredients (APIs). However, due to the size limitation of the milliposts, this applicator has low dosing size, also the steels milliposts can't be retrieved, and will stay in the body, which increases the potential risk. Therefore based on SOMA, Abramson et al. [90] developed a novel orally dosed liquid auto-injector called the liquid-injecting self-orienting millimeter-scale applicator (L-SOMA) (Fig. 5B), which solves the problem of low drug loading of SOMA. And it has the capability of delivering up to 4-mg doses of bioavailable drug with the rapid pharmacokinetics of an injection, reaching an absolute bioavailability of up to 80 % and a maximum plasma drug concentration within 30 min after administration. The core idea of L-SOMA is to use three different springs to release in stages, so as to complete the three processes of injecting a hypodermic needle beneath the gastric mucosa, delivering 80 μL of liquid drug formulation into the submucosal space and retracting the needle into the device after injection. Additionally, Abramson et al. [92] developed a luminal unfolding microneedle injector (LUMI) which allows for the oral delivery of biologic drugs by rapidly propelling dissolvable drug-loaded microneedles into intestinal tissue using a set of unfolding arms (Fig. 5C). And similar to the SOMA, this device is also driven by a spring. *In vivo* studies were also conducted to support the safety and validity of LUMI.

The low absorption rate of polypeptide drugs in the stomach is an obstacle to the oral administration of these drugs. Fortunately, the absorption of orally delivered semaglutide, a glucagon-like peptide-1 analog, coformulated with the absorption enhancer sodium *N*-[8-(2-hydroxybenzoyl) aminocaprylate] (SNAC) in a tablet has been investigated, and the result has shown the potential of orally taken large peptide [160,161]. However, its inability to attach to the tissue results in a short residence time of the tablet and causes the low bioavailability of the drug. Inspired by the thorny-headed worms attach in the gut of their host with the help of cylindrical, globular, and retractable thorny proboscis, Chen et al. [110] developed a dynamic omnidirectional adhesive microneedle microrobot for oral macromolecular drug delivery (Fig. 5F). Once the dynamic omnidirectional adhesive microneedle system (DOAMS) is propelled by the spring to penetrate the gastric mucosa, the DOAMS microneedles autonomously changed their shape, so it can greatly prolong the residence time in the gastric mucosa of the device and contribute to enhance the bioavailability of the drug. Moreover, to overcome the barriers of luminal mucus, Srinivasan et al. [93] developed an orally ingestible, robotic drug delivery capsule called RoboCap that locally clears the mucus layer, enhances luminal mixing, and topically deposits the drug payload in the small intestine to enhance drug absorption (Fig. 5D). This capsule can enhance bioavailability 20- to 40-fold greater in ex vivo and in vivo swine models compared to standard oral delivery.

Except trigging by springs, Sarker et al. [162] reported a swallowable capsule for intestinal drug delivery (SCIDD). It can attach the capsule to the gastrointestinal tract via a tissue attachment mechanism (TAM) and then the suction generate by the negative pressure chamber inside the SCIDD will complete the insertion of drug-loaded microneedle into the gastrointestinal mucosa. Since the device needs to maintain a negative pressure, which brings some difficulty in the precision of manufacturing, and maintain a negative pressure environment for a long time may have some trouble, thus, the device may not be suitable for long-term storage. Additionally, Dhalla et al. [163,164] developed an orally ingestible robotic pill (RP) for drug delivery, which is actuated by gas. The pill is encapsulated in an enteric capsule. After entering the intestinal tract, the folded balloon will be inflated rapidly through acid-based reaction, and then the drug-loaded microneedle will be inserted into the mucosa. Once the microneedle is inserted, the balloon will deflate immediately, to avoid the balloon blocking the intestinal tract. And lots of animal experiments in vivo were conducted to verify the effectiveness of the robotic pill in drug delivery. Different from the microrobot that expands with gas generated by acid-base reaction to realize oral intestinal drug delivery, the microneedle robot made of hydrogel material expands into large needle-like vesicles in intestinal fluid environment, and microneedle repeatedly punctures mucosa in the process of intestinal peristalsis contraction and relaxation to realize drug delivery also can be an excellent method [165]. And the materials used to manufacture the microneedle robot are all biocompatible and biodegradable, which ensures the safety of the microneedle microrobot.

Devices resident in the stomach, also can be called “Gastric Resident Systems”, which can be used for a variety of clinical applications including nutritional modulation, ingestible electronics for diagnosis and monitoring, and gastric-retentive dosage forms for prolonged drug delivery. For some extend-released drugs, limited by the rapid gastrointestinal (GI) transit time, the therapeutic efficacy of these drugs will be reduced. So, it's necessary to develop gastric resident systems for prolonging retention time for drugs in the gastric cavity. Hydrogels offer new opportunities for oral gastric resident systems due to their superior mechanical compliance, swelling and biocompatibility. However, researchers always need to make a trade-off in hydrogels with high swelling ratio, high swelling speed and long-term robustness. Therefore Liu et al. [159] presented a gastric retention hydrogel device that can be ingested as a standard-sized pill, which rapidly expands into large soft sphere upon ingestion and remains stable in the stomach for up a month (Fig. 5G). Huang et al. [166] inspired by the natural morphology of peony pollens, they presented a novel multilobe microparticles (MPs) delivery microrobots for target surface adhesion and durable drug release, and their animal experiments revealed these multilobe MPs showed outstanding performance in treating rats with inflammatory bowel disease (IBD). Different from take the advantage of hydrogels to prolong the residue time in the stomach, Ghosh et al. [94] got the inspiration from the Ancylostoma duodenale which can reside in the human intestine for up to 2 years, they developed multiclawed devices with sharp microtips that can latch onto the mucosal tissue, and they presented the first preclinical evidence that these submillimeter-scale mechanochemical therapeutic grippers can enhance drug release and retention for 24 h in vivo (Fig. 5E). Moreover, by using an oral, ultra-long-acting capsule that dissolves in the stomach and deploys a star-shaped dosage form that releases drug while assuming a geometry, Hayward and Bellinger et al. [167,168] achieved to prolong the residence time to more than a week. For some diseases like tuberculosis (TB), require multi-month courses of daily multigram dose for treatment, so capacity of drug is also an important factor needs to be considered. Verma et al. [169] developed a gastric resident system that was capable of safely encapsulating and releasing grams of antibiotics over a period of weeks to address the challenge of prolonged dosing for regimens requiring multigram drug dosing. Babaee et al. [170] inspired by a blooming flower, using ingested warm fluids act as triggers, degradable milli needles incorporated into the microrobot to deliver the drug molecules. And the gastric-resident platform they developed act as a gram-level long-lasting drug delivery dosage form, releasing small-molecule drugs for 2 weeks.

### 5.3 Self-propelled drug delivery microrobots

**Fig. 6:**
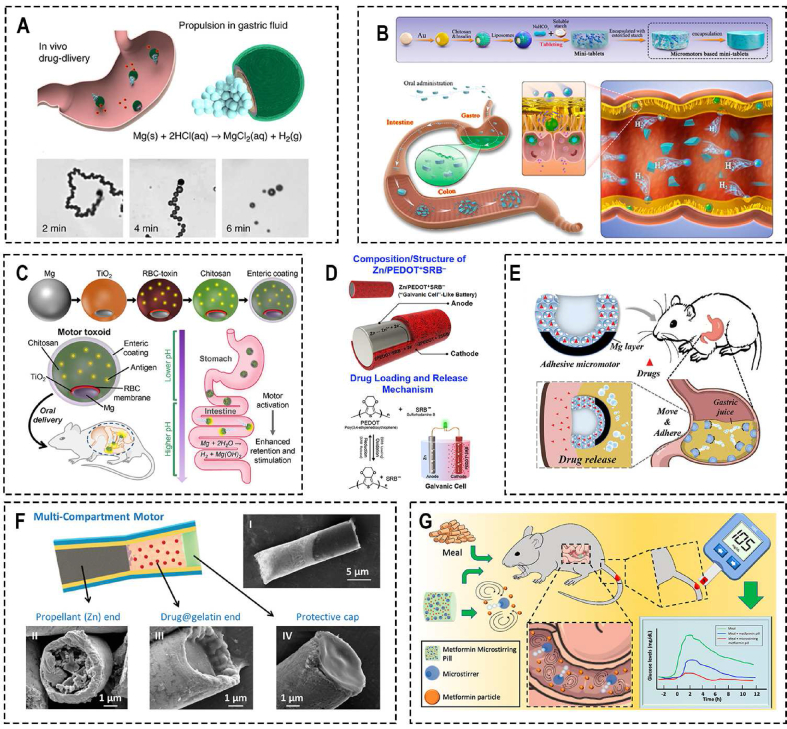
Self-propelled Drug Delivery Microrobots. (A) In vivo therapeutic Mg-based micromotors application loaded with antibiotic drug clarithromycin (CLR) for treatment of *H. pylori* infection in a mouse model [177]***. Copyright ©2017 Springer Nature***; (B) A micromotor based mini-tablet platform with colonic triggered insulin release and active drug delivery for the treatment of diabetes [181]***. Copyright ©2023 American Chemical Society***; (C) Mg-based biomimetic self-propelling micromotors for oral ant-virulence vaccine delivery [179]***. Copyright ©2019 American Chemical Society***; (D) A self-powered battery-driven drug delivery micromotor which comprise an inner rod of zinc and an outer shell of positively charged poly (3.4 ethylenedioxythiophene) [178]***. Copyright ©2019 Elsevier Ltd***; (E) Mg-loaded drug delivery micromotors with the capable of adhering to the intestinal tissue based on the suction-cup-like structure [84]***. Copyright ©2021 Wiley-VCH GmbH***; (F)Multicompartment tubular micromotors for efficient site-specific cargo delivery [182]***. Copyright ©2020 Wiley-VCH GmbH***; (G)A Microstirring oral pill for improving the glucose-lowering effect of metformin [183]***. Copyright ©2023 American Chemical Society***.

Due to the carriers in the gastrointestinal tract, biological therapeutic with poor solubility, poor permeability in the gastrointestinal environment will have poor bioavailability [171], thus the researchers had developed a novel drug delivery microrobot that can achieve self-propelled. Different from magnetic-controlled microrobots, self-propelled microrobots are micromachines which can convert energies into their own mechanical motion. Once they are taken orally, the microrobot firstly travel into the GI tract, adhere, or penetrate the GI mucosa for prolonging the retention time and then release the loaded drugs. The properties of the controlled navigation, propelling force, cargo towed and release, and tissue penetration to prolong the retention time are essential for most drug delivery devices [172]. And for practical applications, the biodegradability and the ability to achieve precise delivery of drugs to target specific section of body are also important aspects that must be taken into consideration, particularly in biomedical field [173]. Since self-propelled microrobots have all the desired properties, including tiny size, autonomous motion, and navigation capacities, they have also shown great potential for applications in the biomedical fields, like drug delivery [174]. As Zn and Mg are both “green” nutrient trace elements, vital for many body functions and metabolic, and they can be redox in acidic environments to produce hydrogen, therefore, these kinds of metal materials are always selected as the fuel material to react with the H^+^ in the gastric environment. Gao et al. [109,175,176] developed a novel chemically powered zinc-based micromotors, which can move at a high speed of ∼60 μm/s and can be used for gastric drug delivery. The self-propulsion in the local stomach environment led to greatly improved tissue penetration and retention time. This micromotor will destroy themselves upon completing their delivery mission. The authors also demonstrated the first study of micromotor under in vivo conditions and characterized the micromotors' distribution, retention, cargo delivery, and toxicity profile in mouse stomach. Later, Berta et al. [177] presented the first in vivo therapeutic Mg-based micromotors application loaded with antibiotic drug clarithromycin (CLR) for treatment of *H. pylori* infection in a mouse model (Fig. 6A). Different from releasing drugs need to dissolve the zinc-based body firstly, Cui et al. [178] inspired by “galvanic cell”, using a Zn core, a PEDOT^+^SRB^−^ shell, and the electrolyte solution form a “galvanic cell”, had developed a battery-driven drug delivery device, which can self-propelled at low pH, and release loaded drugs at higher pH based on two sets of redox reactions (Fig. 6D). In order to maximum the utility of vaccines, Wei et al. [179] used a magnesium-based core as fuel and a biomimetic cell membrane used for detaining and neutralize a toxic antigenic payload to develop a biomimetic self-propelling micromotor formulation (Fig. 6C). And they have confirmed that the micromotor greatly improves the retention and uptake of the antigenic material in the small intestine in vivo. Except for using metal as fuel material, the effervescent tablet is also a good choice. Additionally, it is also a very common drug which can generate lots of bubbles once it touches with the aqueous solution. Thus, Cai et al. [89]inspired by the structure and function of rocket, they designed a novel drug delivery microrobot with scaled-down rocket-like architecture. And they used the reaction of effervescent tablet with intestinal juice as driving source. Nevertheless, in some conditions, such as gastric cancer therapeutic, drugs need to be delivered to the target sites in the stomach to guarantee the efficacy of the treatment. However, those designed self-propelled microrbots can just work in the gastrointestinal environment but can't control micromotors to release the drugs in the desired segments of GI tract. Therefore, to achieve selectively locating therapeutic to specific GI segments. Li et al. [86] demonstrated a biocompatible enteric microrobot that can selectively position in a specific segment of the GI tract and actively penetrate the tissue to prolong the retention time. This microrobot consists of the coating which is stable in acidic conditions but soluble in neutral or alkaline media, and the Mg body that allows for spontaneous propulsion in the intestinal fluid. By simply controlling the coating thickness, it is feasible to selectively activate the propulsion of the microrobot and consequently to determine the location of the GI tract for penetration and retention time. Using the same principle, Maric et al. [180] used Eudragit polymer coating as a pH sensitive layer for microrobot construction to achieve drugs targeted release in the GI tract. *In vitro* drug release study under acidic and neutral pH chemical environments had confirmed the function as pH-sensitive microdevices for drug release. Zhou et al. [91] inserted a thin Fe intermediate layer to the micromotor, thus achieved the navigation to the targeted sites by magnetically controlling. The combination of self-propelled microrobots and selectively positioning enhanced the delivery ability and therapeutic efficacy of the micromotor. Additionally, this combination may also extend the applications of microrobots.

For most drug delivery microrobots rely on self-propulsion to penetrate the tissue and realize to prolong the retention time. Nevertheless, in some complex biological environments, using a substantial adhesion mechanism, rather than penetration can significantly improve the clinical transformation of the drug delivery. Cai et al. [84] insighted the importance of developing micromotors with adhesive properties, and inspired by the suction cup of octopi, they proposed an ingenious micromotor with adhesive property for drug delivery in the stomach. The shape of this micromotor is like the suction cup of octopi, and it achieves spontaneous movements by hydrogen bubble derived from Mg/H^+^ reaction (Fig. 6E). With its special shape and spontaneous movements, the micromotor could move and adhere efficiently to the tissue for drug releasing. Later, they also presented an ingenious hydrogel microparticle based on the mechanism of Boston ivy tendrils adhesive discs [184]. And they have demonstrated that these novel microparticles exhibited prominent adhesive property to the wet tissue environment and can prolong the maintenance of drug availability. Except bubble propulsion, there are also some other propulsion mechanisms, including propelled by ultrasonic field [185] and light field [186].

While the self-propelled microrobot has many advances, and created exacting opportunities for oral administration, further improvements need to be considered. Berta et al. [182] thought that further improvements should focus on the spatial separation of the propulsion and cargo-carrying functions (Fig. 6F). Based on this idea, they designed a multicompartment tubular micromotor that consists of a black-end zinc engine and an upfront cargo compartment protected by a pH-responsive cap. This micromotor can achieve site-specific responsive cargo release and enhanced retention in the gastric tissue. Traditional self-propelled motors are highly reactive to the fuel, and this may cause short propulsion time and poor efficacy, Karshalev et al. [187] combined the advantages of traditional pills with the efficient movement of micromotors, they presented a swallowable micromotor pill which consisted of active Mg-based micromotors dispersed uniformly in the pill matrix. They have demonstrated that the micromotor pill platform effectively protects and carries the active micromotors to the stomach, enabling their release in a concentrated manner. Based on the same idea, Mundaca-Uribe et al. [183,188] used magnesium-based micromotor as micro stirrers and reported a microstirring pill with built-in mixing capability for oral drug delivery that enhances bioavailability of its therapeutic payload and the drug loading capacity (Fig. 6G). In order to improve the tolerance of oral insulin to harsh digestive environment as well as mucosa absorption barrier, Liu et al. [181] constructed the insulin layer onto the surface of a magnesium based micromotor via electrostatic interactions, they developed an insulin-loaded mini-tablet microrobot for oral insulin delivery (Fig. 6B). Additionally, Wu et al. [189] found that existing micro/nanomotor platforms are inefficient for deep issue imaging and motion control in vivo, thus it was hard for precisely targeted delivery using micromotors. To solve this problem, they presented a photoacoustic computed tomography (PACT)-guided microrobotic system (PAMR), in which visualization in real time in vivo is achieved by PACT.

### 5.4 Biohybrid drug delivery microrobots

**Fig. 7:**
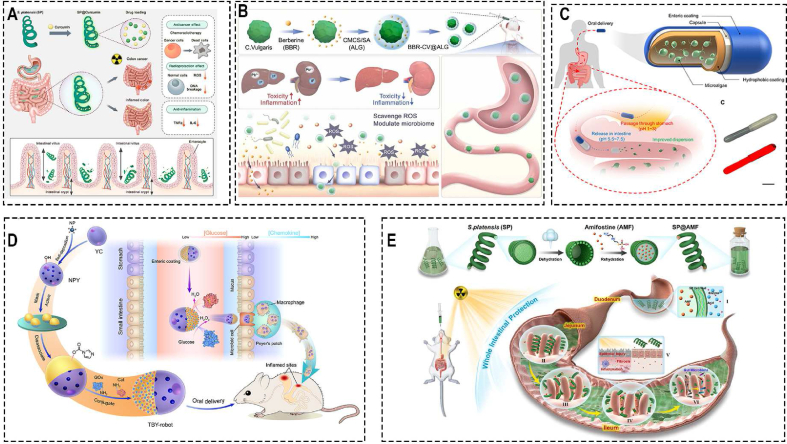
Biohybrid Drug Delivery Microrobots. (A) A versatile formulation based on a helical-shaped cyanobacterium, Spirulina platensis (SP), loaded with curcumin (SP@Curcumin) for the treatment of colon cancer and colitis [190]***. Copyright ©2021 AAAS***; (B) A multifunctional oral, cross-linking microalgal hydrogel system with pH-sensitive biodegradability consisting of Chlorella vulgaris (CVs) and berberine (BBR) for lead poisoning therapy [17]***. Copyright ©2023 Elsevier Ltd***; (C) Efficient algae-based motors embedded inside a pH-sensitive degradable capsule for gastrointestinal tract drug delivery [95]***. Copyright ©2022 AAAS***; (D)A twin-bioengine yeast micro/nanorobot (TBY-robot) with self-propelling and self-adaptive capabilities for gastrointestinal inflammation therapy [79]***. Copyright ©2023 AAAS***; (E) Oral drug delivery microrobots utilizing microalgae Spirulina as a carrier for amifostine for intestinal radiation protection [20]***. Copyright ©2022 Springer Nature***.

Biohybrid oral administration microrobots are a novel type of drug delivery microrobot that combines microorganisms with traditional drug delivery devices. Due to the natural characteristics of some microorganisms, certain natural microorganisms offer better biocompatibility and biodegradability compared to synthetic materials. Moreover, many microrobot designs mimic biological features. Therefore, directly utilizing microorganisms as important components of drug delivery robots is an emerging and highly regarded approach. Zhong et al. [190] focus on the issues of poor therapeutic efficacy, low bioavailability, and biocompatibility of oral drug delivery (Fig. 7A). They used Spirulina platensis (SP) as a carrier to construct a microalgae-based oral drug delivery microrobot (SP@Curcumin) for the treatment of colon cancer and colitis. Self-propelled drug delivery robots suffer from limited onboard fuel, resulting in restricted active mobility and short propulsion time. Microorganisms such as microalgae and bacteria can sustain continuous movement without external intervention. Zhang et al. takes advantage of the fast and long-lasting swimming behavior of natural microalgae in intestinal fluid, they reported an efficient algae-based motor platform to prolong local retention within the gastrointestinal tract (Fig. 7C) [95]. Even more astonishingly, it has been demonstrated that acidophilic microalgae biohybrid microrobot can maintain their swimming behavior over long periods of time in the harsh acidic environment of the stomach, thus enabling them to be applied for gastrointestinal (GI) delivery applications [191]. This demonstrates significant potential for the application of biohybrid drug delivery microrobots in the stomach. In addition, recently, microalgae have also been found to perform well in intestinal radiation protection and lead poisoning therapy. Liu et al. reported a multifunctional and biocompatible oral, cross-linking microalgal hydrogel microrobot with pH-sensitive biodegradability consisting of Chlorella vulgaris (CV) and berberine (BBR) (Fig. 7B) [17]. This microrobot can absorb and remove Pb in the living body and alleviate lead poisoning-related disease. Using microalga Spirulina platensis as a microcarrier of Amifostine to construct an oral delivery microrobot has displayed comprehensive drug accumulation and effective radiation protection within the small intestine and also shows benefits on the gut microbiota homeostasis and long-term safety [20] (Fig. 7E). Combining natural microalgae Spirulina platensis (SP) with astaxanthin nanoparticles (ASXnano) allows for the full utilization of their complementary and synergistic effects. This achieves improvements in the distribution of drugs in the intestines and blood, protects intestinal microbial populations, and ultimately mitigates radiation-induced intestinal and systemic damage [16].

In addition to microalgae, research on the application of probiotics in oral administration is also gaining momentum. Utilizing probiotic spores as drug carriers can overcome spatial variations and multiple biological barriers in the gastrointestinal environment, facilitating the rapid delivery of drugs into the intestinal milieu [111]. The combination of Bacillus spores with curcumin has been found to significantly enhance the oral bioavailability of curcumin, offering a novel approach for the treatment of colon cancer [24]. Yeast cell walls, characterized by hollow and biodegradable porous microspheres, can encapsulate chemical substances and small particles. Zhang et al. [79] introduced a twin-bioengine yeast micro/nanorobot (TBY-robot) with self-propelling and self-adaptive capabilities, autonomously navigating to inflamed sites for gastrointestinal inflammation therapy via enzyme-macrophage switching (EMS) (Fig. 7D). This delivery method has been found to increase drug accumulation in diseased sites by approximately 1000-fold, significantly alleviating inflammation in mouse models of colitis and gastric ulcers and improving disease pathology.

## 6 Clinical translation challenges and prospection

While significant progress has been made in the research domain concerning microscale drug delivery robots, the clinical translation prospections and challenges of these emerging oral delivery strategies and therapies are of considerable concern. Therefore, it is meaningful to discuss the clinical translation of oral drug delivery microrobots.

Firstly, we have summarized the advantages and limitations of each type of oral drug delivery microrobots mentioned above and provided explanations on the challenges and prospects encountered in the clinical translation of oral drug delivery microrobots.

### 6.1 Advantages, limitations of oral microrobots

#### 6.1.1 Magnetic-controlled drug delivery microrobots

Compared to the depth limitation of light propulsion and the thermal and efficiency issues associated with ultrasonic propulsion, magnetic propulsion is a non-invasive and controllable propulsion method. With the continuous development of magnetic driving devices and the improvement of control accuracy, magnetic-controlled drug delivery microrobots can achieve complex and precise movements or specific deformations under external magnetic field driving. This robust mobility and control capability are lacking in other types of drug delivery microrobots. These capabilities enable oral drug delivery microrobots to overcome various gastrointestinal barriers, offering more possibilities for oral therapy, including treatment of hard-to-reach areas and targeted therapy for specific lesions. Magnetic fields can also induce magnetic-controlled drug delivery microrobots to perform hyperthermia therapy, which is beneficial for treating tumors in the gastrointestinal tract. Combined with medical imaging equipment and endoscopes, magnetic-controlled drug delivery microrobots can even overcome compromises in the reachable range caused by highly precise movements and achieve targeted therapy in the bile duct areas of the digestive system that conventional oral therapy cannot reach [96,150]. However, an important challenge of magnetic-controlled drug delivery microrobots is the core magnetic materials required for propulsion. Many reported magnetic drug delivery microrobots contain materials with poor biocompatibility, such as nickel-based and neodymium-based metals and alloys [151]. Currently, iron-based materials with relatively good biocompatibility are the most promising. In addition, since oral therapy with magnetic-controlled microrobots depends heavily on magnetic driving devices, some high-precision magnetic driving devices are expensive. This not only increases the treatment cost of magnetic-controlled drug delivery robots compared to other oral drug delivery microrobots but also complicates the treatment process. For diseases requiring daily oral medication, such as diabetes treated with oral insulin, magnetic-controlled drug delivery microrobots are not significantly competitive compared to anchored drug delivery microrobots, self-propelled and biohybrid drug delivery microrobots. However, for targeted therapy in the gastrointestinal tract and treatment in areas such as the bile duct in the digestive system, magnetic-controlled drug delivery microrobots can maximize their advantages.

#### 6.1.2 Anchored drug delivery microrobots

These microrobots can anchor their body or drug-carrying components in the gastrointestinal tract, mainly used for the treatment of orally administered macromolecules or biologics, or for long-term continuous oral administration. Moreover, the entire drug delivery process of these microrobots, from ingestion to excretion, relies almost entirely on internal mechanisms, making the operation straightforward and similar to conventional oral capsule intake. Furthermore, anchored drug delivery microrobots can effectively overcome mucosal barriers in the gastrointestinal tract through clever design of driving modules, significantly enhancing the bioavailability of drugs. For instance, L-SOMA designed by Langer et al. achieved up to 80 % absolute drug bioavailability [90]. Additionally, compared to self-propelled or biohybrid drug delivery microrobots, anchored drug delivery microrobots can achieve higher drug payloads. Anchored drug delivery microrobots generally lack or only have very limited active mobility and complex deformation capabilities. They almost do not possess targeted therapy capabilities within the gastrointestinal tract and are unable to perform complex actions or tasks. These robots are typically sized at around the centimeter scale, which may result in a higher risk of gastrointestinal obstruction when orally administered.

#### 6.1.3 Self-propelled drug delivery microrobots

In oral drug delivery, self-propelled drug delivery microrobots can convert chemical energy into mechanical energy, thereby gaining limited active mobility. Utilizing this limited active mobility, self-propelled drug delivery microrobots can effectively overcome barriers in the gastrointestinal tract, thereby increasing drug residence, penetration, and absorption [181]. Compared to anchored and magnetic-controlled drug delivery microrobots with relatively complex structural designs, self-propelled drug delivery microrobots have simpler structures and smaller sizes. Moreover, since most of these drug delivery microrobots use their body materials as fuel, they leave smaller proportion of residue after completing drug delivery tasks, resulting in lower risk of intestinal obstruction compared to other drug delivery microrobots. However, the smaller size also means limited fuel capacity for these microrobots, limiting the generated propulsive force and duration of action. Additionally, the process of converting chemical energy into mechanical energy is mostly based on chemical reactions, and some rapid reaction processes make self-propelled drug delivery microrobots generate a large amount of driving force in a short period, making the movement process uncontrollable. Currently, there is still significant demand for improving efficiency, extending propulsion lifetime, adaptability, and biocompatibility for self-propelled drug delivery microrobots [106].

#### 6.1.4 Biohybrid drug delivery microrobots

Biohybrid drug delivery microrobots consist of a combination of biological organisms and synthetics, providing an attractive strategy for achieving biomimetic behavior and advanced functionality. Utilizing microorganisms as the primary raw material, biohybrid drug delivery microrobots have better biocompatibility compared to other drug delivery microrobots. Moreover, these microrobots can generate strong and persistent thrust for self-propulsion without external driving, perceiving changes or stimuli in the surrounding environment and reacting to them, which are characteristics that other types of drug delivery robots lack [19]. Furthermore, due to the unique natural properties of different microorganisms, rational utilization of these properties can endow oral drug delivery microrobots with stronger functionality, including inherent tumor targeting and penetration properties of some bacteria [193], protection of loaded drugs from degradation under harsh conditions, prolonged intestinal retention effects, and wide availability and low cost of microalgae or bacterial materials [20,190]. In addition to utilizing the inherent properties of microorganisms, decorating microorganisms with different functional units can further expand the application scenarios of biohybrid drug delivery microrobots, creating multifunctional oral drug delivery microrobots [191].

One of the challenges of using biohybrid drug delivery microrobots is their controllability. The common phototacticity of microorganisms cannot be controlled in the oral field because visible light cannot penetrate deep tissues, making it impossible to control their movement through this method [19]. Additionally, although various in vitro studies have proven the use of bacteria-based drug delivery microrobots, their use in vivo poses certain risks when considering factors such as pathogenicity or immunogenicity [95].

### 6.2 Safety

For oral drug delivery microrobots, drug therapy is the critical goal. More than 90 % of drug candidates fail during clinical trials for lacking efficacy and poor material properties, and ∼30 % of drug candidates fail clinical testing owing to unmanageable toxicity [151]. Therefore, safety is the key factor for the real application of these drug delivery robots in clinical treatment. Here we will evaluate the safety from the following two aspects: the size and shape of the microrobots, and the biodegradability and biocompatibility of the materials.

Since the general oral treatment requires delivery of drugs to the stomach or small intestine, it is inevitable to pass through the esophagus or small intestine, but the diameters of the esophagus and small intestine are small (small intestine diameter 2.5–3 cm). These limitations on the structure of human gastrointestinal tract have a very important impact on the safety of oral drug delivery microrobots. The size and shape of pharmaceutical products can affect product transportation through the pharynx and esophagus and may directly affect patients’ ability to swallow a particular pharmaceutical product. Larger pharmaceutical products have been shown to have prolonged esophageal transit times, which may cause the product to disintegrate in the esophagus and/or cause esophageal damage, resulting in pain and localized esophagitis, and may produce serious sequelae including ulcers, perforation, and vomiting [[194], [195], [196]]. Beyond that, larger sizes also carry the risk of intestinal obstruction. Therefore, when designing drug delivery robots, the overall size and shape of the device must be limited for the safety of treatment. The US Food and Drug Administration (FDA) approved Daily Drug delivery Osmotic Controlled Release Oral Delivery System (OROS) (φ9mm×15mm), a non-degradable drug delivery capsule with obstruction rates of 1 in 29 million [197], provides a dimensional reference for the design of other oral delivery microrobots. For example, in the anchored drug delivery microrobot introduced in Section 5, the size of SOMA is 10 mm in diameter and 12 mm in height, while LUMI is 9 mm in diameter and 30 mm in height. For oral treatment, as the result of the gastrointestinal transit time and retention risk rapidly increasing with the increase of the device size, the biocompatibility of the device and the risk of obstructing gastrointestinal tract caused by the retention of the device cannot be ignored. Therefore, to reduce the device in the volume of non-biodegradable parts as far as possible, using some materials that is biocompatible and biodegradable is very important [198]. Biocompatible and biodegradable materials have been mentioned in Section 3 and thus will not be discussed here. Ideally, biodegradable solids that remain in the gastrointestinal tract need to be degraded within 24 h to ensure there is no risk of clinical obstruction [199]. It should be noted that biodegradable materials do not necessarily imply that microrobots are non-toxic. The polymer structures, polymer subunits, or chemical degradation products of biodegradable microrobots may cause toxicity [200]. Even if microrobots do not cause immediate reactions, the accumulation of subunits and degradation by-products in certain organs, especially the liver, spleen, and kidneys, may lead to irreversible metabolic disorders over the long term. Therefore, the toxicity of biomedical microrobots should be tested in live tissues for up to 12 months, including all individual components of the entire device and throughout the degradation process [200].

In addition, the wall thickness of the stomach is 4–6 mm and the wall thickness of the small intestine is 0.1–2 mm. For microneedle drug delivery robots, it is necessary to insert the microneedle into the mucosa to release the drug, but it is needed to prevent the adverse effects of perforation. Fortunately, TRAVERSO et al. [201] using a model that exposed radially protruding microneedles, demonstrated that a capsule coated in 25-gauge metal microneedles protruding 5 mm from a device could pass through the gastrointestinal tract without evidence of mucosal damage. This study provides theoretical support for the safety of anchor drug delivery microrobots.

In conclusion, safety is the most important attribute of pharmaceutical products, and the design of oral drug delivery microrobots should ensure that the robot will not have adverse effects on the human body. Non-toxic, degradable, and biocompatible materials should be selected as far as possible. The size of the device should also be reduced as far as possible under the condition that it can carry enough therapeutic drugs, and sharp structures should be reduced in appearance.

### 6.3 Challenges and prospectives in clinical translation

The maturity of a new technological design can be assessed through its Technology Readiness Level (TRL). TRL quantifies the progress of an emerging technology through its stages of research, development, and implementation, ranging from 1 to 9, where 9 represents the most mature technology. For medical devices, TRL 9 signifies distribution and marketing for clinical use. Currently, many oral drug delivery microrobots have completed many experimental validations of conceptual models and conducted some in vivo drug loading experiments, a design of this nature may attain TRL 5 (concept validation) [200]. But drug development should be aimed at eventual clinical translation, therefore, oral drug delivery microrobots should also continue to evolve towards this goal. Here, we discuss the challenges in clinical translation of oral drug delivery microrobots.

The high controllability and precision mobility demonstrated by magnetic-controlled drug delivery robots have attracted great attention in the field of oral administration, especially in oral targeted therapy. In recent years, a large number of research studies have emerged. However, regardless of whether the safety issues are based on magnetic driving devices or the materials of microrobots, the current research is mostly in the in vitro stage, with a few studies conducting in vivo experiments in animals. At present, there is a lack of oral drug delivery microrobot performance in animal models. To achieve true clinical translation in the future, the next step should focus more on the treatment performance of magnetic-controlled drug delivery microrobots in animal models, continuously monitor relevant physiological indicators during treatment, and focus on the safety of core magnetic materials of magnetic-driven drug delivery robots in vivo. Currently, there are variations in the magnetic actuation devices used in the research of magnetic-controlled drug delivery robots. Clinical magnetic resonance imaging (MRI) scanners typically have magnetic fields ranging from 1.5 to 3 T (T), while 7-T systems have recently been approved by FDA for clinical use in the United States. Some commercially available MRI platforms can generate magnetic field gradients of ≥24 T/m [202]. Before moving towards clinical translation, it is essential to consider achieving precise control of magnetic-controlled drug delivery microrobots under clinically permitted magnetic field conditions and to address compatibility issues between magnetic actuation devices and medical equipment used in hospitals and clinical environments [151]. To further improve targeted therapy effectiveness and control precision, integrating real-time imaging systems into the magnetic actuation system for auxiliary control could be considered [202]. Extensive research on the safety of magnetic actuation devices for clinical practitioners and patients is also necessary. Magnetic actuation devices should not pose any potential risks to patients and clinical practitioners, thus laying the groundwork for future clinical practical applications [151].

On the front of clinical translation, anchored drug delivery microrobots have already demonstrated promising therapeutic effects in large animals such as pigs [87,165]. With the integration of advanced manufacturing technologies, the production cost of these robots can be controlled within several dollars. Combined with their independence from external assistance during drug delivery, there are reasons to believe that anchored drug delivery microrobots have greater prospects for the treatment of diabetes with oral insulin or other macromolecular drugs compared to other types of oral delivery microrobots. The next step will involve conducting corresponding clinical trials to evaluate patient responses to the anchor delivery microrobots. However, to achieve true clinical translation, long-term repeated dosing experiments in preclinical and clinical settings are still needed. These trials should include patients of different ages, weights, heights, diets, and disease conditions, while monitoring any adverse reactions, including pain and tissue damage, especially damage to organ walls [90]. Additionally, establishing a unified, standardized, and compliant manufacturing process to ensure the quality and safety of produced robots is also crucial for these drug delivery robots to consider.

Currently, self-propelled and biohybrid drug delivery microrobots based on microalgae, bacteria, and other microorganisms have conducted extensive research in rodent animal models and achieved certain therapeutic effects. However, rodents are not phylogenetically related to humans, and their genetics, body size, and lifespan differ significantly from humans [181]. Compared to rodents, large animal models such as pigs and dogs have many similarities with humans in terms of anatomical structure, physiological metabolism, and disease mechanisms [190]. Therefore, on the path to clinical translation, the first thing to address is whether these drug delivery robots can still achieve good and stable therapeutic effects in large animal models, especially in non-human primate models, by conducting systematic safety and efficacy assessments. Furthermore, long-term safety monitoring data should be available to assess the safety and immunogenicity of biohybrid drug delivery microrobots using microbial materials in the complex microbial environment of the human gastrointestinal tract before conducting clinical studies. Selecting appropriate microbial carriers for specific disease models and optimizing the drug loading process are also issues that need to be addressed before clinical translation [190].

Here, we further discuss the clinical translation of oral drug delivery microrobots from the perspectives of manufacturing processes and industrialization.

Regarding the various processes of design and manufacturing, in terms of material selection for oral drug delivery robots, it is advisable to choose materials approved by regulatory agencies such as the FDA or equivalent organizations. This helps ensure the safety of the designed and manufactured products and increases the likelihood of obtaining approval from the corresponding regulatory authorities. Manufacturing processes should also consider drug contamination, manufacturing efficiency, and cost. Additionally, for oral medications, the impact of food on bioavailability should be studied, which is particularly important for drugs that may alter release behavior. Typically, in single-dose pharmacokinetic studies, an appropriate dose should be selected for studying the effects of food on drug absorption.

In summary, various oral drug delivery microrobots have shown promising results in the treatment of specific oral diseases. However, there are still many shortcomings. Clinical translation should be guided by the ability to obtain approval from regulatory agencies and requires the establishment of unified, standardized, and compliant design and manufacturing processes [11].

## 7 Conclusions and future perspectives

Oral drug delivery microrobots hold tremendous potential in oral administration. A lot of in vivo and in vitro experiments has demonstrated that such microscale robots can more effectively overcome gastrointestinal barriers, enhance the bioavailability of biologics drugs, improve drug targeting capabilities, and expand the applicability of oral therapy. Compared to traditional oral medications, which face challenges such as low bioavailability, complex manufacturing processes, and higher costs, with limited applicability, oral drug delivery microrobots have significantly improved the feasibility of oral administration and advanced precise targeted treatment. Microrobots typically eliminate the need for complex drug processing, especially in the case of biologics like oral insulin. Through smart structural design, these microrobots can greatly enhance the bioavailability of medications, thereby reducing costs associated with such therapies. Moreover, with the increasingly mature technology of 3D printing or other advanced manufacturing techniques, mass industrialization of microrobots is foreseeable.

Microrobots possess diminutive sizes ranging from hundreds of micrometers to centimeters, enabling them to navigate the human gastrointestinal tract similar to capsule ingestion. Tailored microrobots with different sizes and propulsion mechanisms can be designed for various disease scenarios. For oral biologic therapy, patients can ingest microrobots without the need for external driving devices, similar to regular capsule ingestion. This eliminates the discomfort associated with injections, particularly beneficial for conditions like diabetes. For microrobots requiring external control, magnetic field propulsion is widely employed, ensuring minimal patient discomfort and non-invasive treatment. Particularly in gastrointestinal cancer treatment, microrobots can precisely deliver drugs to the lesion area under magnetic control, minimizing side effects from treatments like radiotherapy and chemotherapy, thus mitigating patient harm. Therefore, microrobots offer significant advantages and potential in simplifying drug administration, enhancing patient compliance, and reducing treatment discomfort.

Despite the achievements made in oral drug delivery microrobots, numerous challenges persist. Firstly, there is a contradiction between the size and payload of oral drug delivery microrobots and their functionality. Particularly for anchored drug delivery robots, reducing the size of the robot can mitigate the risk of intestinal obstruction but may lead to a reduction in drug payload and functional modules. Additionally, oral drug delivery microrobots typically involve a variety of complex materials, raising concerns about material biocompatibility, biodegradability, and long-term safety issues that need to be addressed prior to clinical translation. Moreover, oral drug delivery microrobots are still in the laboratory research stage, and for these microrobots to transition from the lab to the clinic, innovation must align with clinical needs and real-world healthcare practices [151]. Furthermore, as an emerging frontier in research, there is a lack of established regulations regarding the clinical translation of oral drug delivery robots. Relevant regulatory agencies should enact regulations and legislation to ensure safety, feasibility, effectiveness, and interoperability [200]. Currently, apart from some anchored drug delivery microrobots that have undergone corresponding studies in large animal models such as pigs and dogs, research on magnetic-controlled, self-propelled, and biohybrid drug delivery microrobots in large animal models is relatively scarce. Before entering clinical trials, extensive animal testing is required to evaluate the biocompatibility, safety, and pharmacology of drug delivery microrobots.

From a broader perspective of clinical and industrial transformation, transitioning laboratory products to the market faces many challenges. For research purposes, the design and manufacture of microrobots are typically conducted in sterile laboratories and do not involve large-batch production. This ensures that the produced products are not contaminated, and batch-to-batch variations are controlled. However, for clinical and industrial transformation, factors such as material safety and procurement, reliability and efficiency of manufacturing processes, differences between batches, quality control, storage, and final waste disposal are crucial considerations [11]. Considering the perspectives of patients and the market, the final price of the product and the comfort of the treatment process are also key considerations. To address these challenges, the following factors should be considered in the initial design of oral drug delivery microrobots: systematically considering the source, price, and properties of materials; designing appropriate controls and driving methods; adopting manufacturing methods that are easy to automate and scale; conducting relevant animal experiments in a timely manner; and focusing research efforts on core clinical concerns. What's More, standardized development stages should be established, including engagement with medical professionals and healthcare markets to identify clinical needs and design task-oriented systems that can iterate towards clinical translation. The ethical, social, and regulatory implications of medical delivery microrobots should be considered throughout their entire product lifecycle, including experimental design in animal studies, informed consent for clinical research, patient bodily integrity and control, conflicts of interest, and potential long-term risks. Animal experiments and clinical trials must be ethically justified, with careful consideration given to their design [200].

To conclusion, oral drug delivery microrobots have shown us a drug delivery method different from traditional oral drugs, weakening the dependence of oral therapy on drugs, and greatly enhancing the universality of oral administration. With the continuous development of disciplines such as pharmacy, control theory, mechanics, computer science, and materials science, we have reasons to believe that microrobots will bring about a significant revolution in traditional pharmaceutical science in the future, continuously driving the development of oral administration and making oral administration a more widespread and effective treatment method.

## 8 Ethics approval and consent to participate

Not applicable.

## CRediT authorship contribution statement

**An Ren:** Writing – original draft. **Jiarui Hu:** Validation. **Changwei Qin:** Validation. **Neng Xia:** Writing – original draft, Validation, Conceptualization. **Mengfei Yu:** Validation, Conceptualization. **Xiaobin Xu:** Validation, Conceptualization. **Huayong Yang:** Resources. **Min Han:** Validation, Conceptualization. **Li Zhang:** Writing – review & editing, Supervision. **Liang Ma:** Writing – review & editing, Supervision.

## Declaration of competing interest

The authors declare no conflict of interest.

## ABBREVIATIONS

3D: Three dimensions
5-FU: 5-fluorouracil
AMF: Alternating magnetic field
API: Active pharmaceutical ingredient
ASXnano: Astaxanthin nanoparticles
BBR: Berberine
CADs: Computer-Aided Design
CLR: Clarithromycin
CVs: Chlorella vulgaris
DCCMHR: Double curved conical micro-helical robot
DHM: Degradable hyperthermia microrobot
DLP: Digital light processing
DOAMS: Dynamic omnidirectional adhesive microneedle system
DTT: Dithiothreitol
ECM: Extracellular matrix
EE: Enteric elastomer
EMADIS: Endoscopy-assisted Magnetic Actuation with a Dual Imaging System
EMS: Enzyme-macrophage switching
FDA: Food and Drug Administration
FDM: Fused deposition modeling
GelMA: Methacrylate gelatin
GI: Gastrointestinal
GLP-1: Glucagon-like Peptide-1
HA: Hyaluronic acid
IBD: Inflammatory bowel disease
L-SOMA: The liquid-injecting self-orienting millimeter-scale applicator
LUMI: Luminal unfolding microneedle injector
M: cells Microfold cells
MMR: Modular microrobot
MN: Microneedle
MNP: Magnetic nanoparticle
MOF: Metal-organic framework
MOFBOT: MOF-based small-scale robot
MOFs: Metal-organic frameworks
MP: Microparticle
MRI: Magnetic resonance imaging
MSCSM: Magnetic stem cell spheroid microrobot
MSNs: Mesoporous silica nanoparticles
NIR: Near-infrared
OROS: Osmotic Controlled Release Oral Delivery System
PACT: Photoacoustic computed tomography
PAMR: Photoacoustic computed tomography-guided microrobotic system
PCL: Poly(ε-caprolactone)
PDMS: Polydimethylsiloxane
PEG: Polyethylene glycol
PEGDA: Polyethylene Glycol Diacrylate
PLA: Polylactic acid
PLGA: Poly (lactic-*co*-glycolic acid)
PSNs: Porous silicon nanoparticles
PVA: Poly (vinyl alcohol)
QDs: Quantum Dots
RIEMS: Rocket-inspired effervescent motors
RMF: Rotating magnetic field
RoboCap: Robotic mucus-clearing capsule
RP: Robotic pill
SCIDD: Swallowable capsule for intestinal drug delivery
SCOD: Suction cup orifice design
SLA: Stereolithography
SNAC: Sodium *N*-[8-(2-hydroxybenzoyl) aminocaprylate]
SOMA: Self-orienting millimeter-scale applicator
SP: Spirulina platensis
SPION: Self-assembled superparamagnetic iron oxide nanoparticle
TAM: Tissue attachment mechanism
TB: Tuberculosis
TBY-robot: Twin-bioengine yeast micro/nanorobot
T: Tesla
TCMR: Triple-configurational magnetic robot
TPP: Two-photon polymerization
TTH: Triggerable tough hydrogel
UV: Ultraviolet
VAM: Volumetric Additive Manufacturing
WMM: Wired magnetic microrobot
YPs: Yeast particles
